# Immunoinformatics Approach to Design a Chimeric CD70-Peptide Vaccine against Renal Cell Carcinoma

**DOI:** 10.1155/2024/2875635

**Published:** 2024-01-27

**Authors:** Haideh Namdari, Farhad Rezaei, Fatemeh Heidarnejad, Mohammad Yaghoubzad-Maleki, Maryam Karamigolbaghi

**Affiliations:** ^1^Iranian Tissue Bank and Research Center, Tehran University of Medical Science, Tehran, Iran; ^2^Virology Department, School of Public Health, Tehran University of Medical Sciences, Tehran, Iran; ^3^Biotechnology Research Center (BRC), Pasteur Institute of Iran, Tehran, Iran; ^4^Division of Biochemistry, Department of Animal Biology, Faculty of Natural Sciences, University of Tabriz, Tabriz, Iran

## Abstract

Renal cell carcinoma (RCC) accounts for the majority of cancer-related deaths worldwide. Overexpression of CD70 has been linked to advanced stages of RCC. Therefore, this study aims to develop a multiepitope vaccine targeting the overexpressed CD70 using immunoinformatics techniques. In this investigation, in silico multiepitope vaccines were constructed by linking specific CD70 protein epitopes for helper T lymphocytes and CD8^+^ T lymphocytes. To enhance immunogenicity, sequences of cell-penetrating peptide (CPP), penetratin (pAntp), along with the entire sequence of tumor necrosis factor-*α* (TNF-*α*), were attached to the N-terminal and C-terminal of the CD70 epitopes. Computational assessments were performed on these chimeric vaccines for antigenicity, allergenicity, peptide toxicity, population coverage, and physicochemical properties. Furthermore, refined 3D constructs were subjected to a range of analyses, encompassing structural B-cell epitope prediction and molecular docking. The chosen vaccine construct underwent diverse assessments such as molecular dynamics simulation, immune response simulation, and in silico cloning. All vaccines comprised antigenic, nontoxic, and nonallergenic epitopes, ensuring extensive global population coverage. The vaccine constructs demonstrated favorable physicochemical characteristics. The binding affinity of chimeric vaccines to the TNF receptor remained relatively stable, influenced by the alignment of vaccine components. Molecular docking and dynamics analyses predicted stable interactions between CD70-CPP-TNF and the TNF receptor, indicating potential efficacy. In silico codon optimization and cloning of the vaccine nucleic acid sequence were accomplished using the pET28a plasmid. Furthermore, this vaccine displayed the capacity to modulate humoral and cellular immune responses. Overall, the results suggest therapeutic potential for the chimeric CD70-CPP-TNF vaccine against RCC. However, validation through *in vitro* and *in vivo* experiments is necessary. This trial is registered with NCT04696731 and NCT04046445.

## 1. Introduction

Renal cell carcinoma (RCC), a type of kidney cancer, accounts for over 90% of kidney-associated malignancies [1]. Managing RCC remains a significant challenge, particularly concerning its metastatic form [2]. The advancements in immunotherapy have led to the approval of drugs aimed at combating advanced RCC [1, 3–6]. However, treatment outcomes exhibit considerable variability [4–6]. Several factors contribute to this variability, including inappropriate adjuvants [3, 4], tumor cell heterogeneity [5, 6], antigen loss within tumors [7], diminished MHC expression [8], inadequate T cell infiltration in tumor tissues [9], and immune suppression via dysfunctional T cells [10]. Notably, the clinical realm has witnessed a surge in antigen-focused T cell therapies for advanced RCC, leveraging immunotherapeutic techniques like peptide-based vaccines [11] to enhance antitumor effects while mitigating adverse impacts. Designing a vaccine for RCC presents multifaceted challenges rooted in the complex nature of this malignancy [12]. Tumor-specific antigens and immune evasion strategies by cancer cells hinder effective immune response elicitation. Additionally, the tumor microenvironment's immunosuppressive nature often thwarts vaccine-induced immune reactions [12]. Moreover, the variability in tumor mutational landscape and the interplay of host factors further complicate efforts to devise a universally effective vaccine. These challenges necessitate innovative strategies to surmount the hurdles in creating a potent and broadly applicable vaccine against RCC. Overlapping or multiepitope peptide sequences recognized by both CD8^+^ and CD4^+^ T cells may help to overcome challenges like tumor heterogeneity, tumor antigen downregulation, and varied HLA haplotypes [4].

Several clinical investigations have aimed at distinct tumor-associated antigens in individuals with RCC. While these studies demonstrated certain favorable advancements, their outcomes revealed only partial responses when compared to the existing treatments [3–10, 13–15]. Lately, there has been a growing focus on directing efforts toward the CD70 molecule in RCC. CD70 serves as a tumor-associated antigen, exhibiting high expression across various malignancies [16–18], such as RCC [19]. CD70, a type II transmembrane protein belonging to the TNF family, is typically expressed on antigen-presenting cells. However, in T and B cells, this molecule is inducible and becomes expressed upon activation [20]. The interaction between CD70 and its corresponding receptor CD27 plays a crucial role in modulating the immune response, regulating activation, and governing proliferation after antigen stimulation [21]. Studies have suggested that the presence of CD70 in RCC tumors correlates with the reduced survival rates [20]. This observation underscores the rationale behind choosing CD70 as a suitable target for RCC immunotherapy. A current clinical trial (NCT04696731) is assessing the safety and effectiveness of CAR-T cell therapy directed against CD70 in RCC [20, 22, 23].

Research into peptide-based cancer vaccines has been thorough, yet their immunogenicity remains moderate. Efforts are underway to explore various strategies aimed at enhancing the efficacy of these vaccines [24]. One way to tackle the inadequacy in the immune response is by combining an adjuvant with the vaccine antigen [25, 26]. Cytokines have emerged as promising adjuvants due to their safe biomaterial nature, derived from humans, and their ability to modulate the immune system [27, 28]. Tumor necrosis factor-*α* (TNF-*α*) is a critical proinflammatory cytokine primarily produced by macrophages and T lymphocytes, playing a pivotal role in both the innate and adaptive immune responses [29]. TNF-*α* cytokine plays a significant role in multiple stages of the immune process, including the activation of the innate immune system, maturation and recruitment of dendritic cells (DC), facilitation of T cell activation, and clearance of pathogens [28]. Many studies across various model systems have highlighted the adjuvant properties of TNF-*α* in combating viral infections [30–32]. One explored method to enhance the efficacy of peptide vaccines involves conjugating antigens with cell-penetrating peptides (CPPs). CPPs are linear peptides possessing cationic and amphiphilic traits. Studies suggest that antigen-CPP conjugates have a heightened capacity to stimulate the immune system compared to the unbound peptides [24]. CPPs aid in facilitating the uptake of the vaccine into the lymphatic system and extending its life span *in vivo* [24]. Studies conducted *in vitro* have demonstrated the ability of CPPs to promote the uptake of antigen peptides by DCs, elevate vaccine immunogenicity in animal models like non-human primates, and improve the efficacy of cancer vaccines in mice, as supported by multiple research endeavors [33–41]. Current clinical trials (clinicaltrials.gov: NCT04046445) are actively evaluating the efficacy of antigen-CPP vaccines. *In vitro* studies have highlighted that coupling with the potent cell-penetrating peptide penetratin (pAntp) enhances antigen uptake and presentation by DCs [42, 43]. Based on these rationales, this study aims to develop a peptide vaccine incorporating CD70, TNF-*α* and CPP penetratin.

## 2. Methodology

### 2.1. Protein Sequence Retrieval

The reference protein sequences for CD70 (NP_001317261.1 CD70 antigen isoform 2 (*Homo sapiens*)) and TNF-*α* (NP_000585.2 (*H. sapiens*)) were retrieved from the NCBI database (https://www.NCBI.com). Additionally, the protein sequence of CPP penetratin was obtained from a previous study [24].

### 2.2. Prediction of Immunogenic Epitopes of CD8^+^ and CD4^+^ T Cells

The web server NetMHCpan-4.1 (https://services.healthtech.dtu.dk/service.php?NetMHCpan-4.1) was configured to predict 9 mer through 14 mer epitopes for HLA class I alleles that were expressed by CD70. The default threshold parameters of 0.5 and 2 were applied to categorize high and low affinity for MHCI binders. The NetMHCpan-4.1 predicts epitopes for 12 HLA class I supertypes, wherein each supertype denotes a group of closely associated HLA alleles with shared affinities for specific peptides due to similar structural traits in the HLA peptide-binding grooves [26]. The web version of NetMHCIIpan-4.0 (https://services.healthtech.dtu.dk/service.php?NetMHCIIpan-4.0) was utilized to predict MHCII binders. By default, epitopes with a length of 15 mers were chosen, and thresholds of 1 and 5 were applied to distinguish high and low affinities, respectively.

Vaccine components must exhibit high antigenicity without triggering adverse reactions. Therefore, in the next phase chosen peptides were filtered based on antigenicity, allergenicity, and toxicity using Vaxijen (https://www.ddg-pharmfac.net/vaxijen/VaxiJen/VaxiJen.html), AllerTOP v. 2.0 (https://www.ddg-pharmfac.net/AllerTOP/), and ToxinPred (https://webs.iiitd.edu.in/raghava/toxinpred/) servers with default settings. Epitopes meeting the criteria of high antigenicity, nonallergenicity, and nontoxicity to the human were selected for further vaccine development phases.

### 2.3. MHC-I Processing Prediction

The NetCTLpan 1.1 server (https://services.healthtech.dtu.dk/services/NetCTLpan-1.1/) was employed to assess the essential stages in MHC class I processing. NetCTLpan 1.1 processes protein sequences as input in 8–11 mer peptides, analyzing their binding with MHC class I, assessing proteasomal C-terminal cleavage, and considering TAP (transporter associated with antigen processing) transport. The threshold default values for the parameters were applied in the current study.

### 2.4. Selection of Cytokine Inducing Epitopes

IFN-*γ* holds a critical role in initiating cellular immunity and subsequently triggering an antitumor immune response [44]. The selected HTL epitopes from the CD70 protein were inputted into the IFNepitope server (http://crdd.osdd.net/raghava/ifnepitope/predict.php) [45] to predict their ability in inducing IFN*γ*. The prediction employed the “Motif and SVM hybrid” methods, with the prediction model set to “IFN*γ* versus non-IFN*γ*” and the submission was made accordingly.

### 2.5. Population Coverage Analysis

Ensuring widespread population coverage is another crucial element in designing vaccines [46]. Variations in HLA types occur among global populations, influencing the antigens presented to HLA molecules. To evaluate the vaccine's coverage across diverse populations worldwide, the IEDB resource's population coverage analysis module (http://tools.iedb.org/population/), with default parameters was utilized. This analysis encompassed MHC-I and -II coverage across different regions, countries, and ethnic groups.

### 2.6. Peptide-Protein Flexible Docking

Assessing the potential formation of MHC-peptide complexes was done using the GalaxyPepDock server (http://galaxy.seoklab.org/cgi-bin/submit.cgi?type=PEPDOCK). Specifically, the binding interactions of each selected CD70 epitope with various MHC alleles were examined. The PDB IDs corresponding to HLA alleles, sourced from the RCSB database (https://www.rcsb.org), were listed in Tables 1 and 2.

### 2.7. Multiepitope Peptide Constructs

We employed top-scoring CTL and Th epitopes to create the final multiepitope vaccine designs. These epitopes were connected via the AAY proteolytic linker sequence to construct chimeric forms. Six peptide constructs were developed utilizing SnappGene®3.2.1, encompassing T cell epitopes sourced from CD70, linked to both the N-terminal and C-terminal regions of the CPP epitope, along with the complete sequence of TNF-*α*, as an adjuvant. Additionally, a construct containing the entire sequence of TNF-*α* was established as a reference for comparative analysis with the other constructs.

### 2.8. The Physicochemical Parameters

The ExPASy-ProtParam tool (https://web.expasy.org/protparam/) was utilized to assess the physicochemical properties of the chosen proteins. ProtParam is a tool that enables the calculation of various physical and chemical parameters for a protein stored in Swiss-Prot or TrEMBL, or for a protein sequence provided by the user. Parameters such as molecular weight, theoretical protrusion index (PI), amino acid, and atomic composition, extinction coefficient, estimated half-life, instability index, aliphatic index, and grand average of hydropathicity (GRAVY) were computed. Other characteristics of the chimeric vaccine constructs were evaluated using various servers. Vaxi-Jen (https://www.ddg-pharmfac.net/vaxijen/VaxiJen/VaxiJen.html) and AllerTOP 2.0 (https://www.ddgpharmfac.net/AllerTOP/) servers were utilized to determine the antigenicity and allergenicity, respectively. Furthermore, the ToxinPred server (https://webs.iiitd.edu.in/raghava/toxinpred/design.php) was employed to predict the toxicity levels of the vaccines [47].

### 2.9. Tertiary Structure Prediction, Refinement, and Validation

To create the tertiary structure of our final vaccines, Rozettafold program in Robetta server (https://robetta.bakerlab.org/) was applied. The GalaxyRefine 2 Server (http://galaxy.seoklab.org/cgi-bin/submit.cgi?type=REFINE2) was employed to modify and optimize the 3D structures according to the key parameters. Additionally, validation was confirmed using ERRAT (https://saves.mbi.ucla.edu/) [48] and verify 3D (https://saves.mbi.ucla.edu/) [49]. Moreover, the PROCHECK server (https://saves.mbi.ucla.edu/) was utilized to generate the Ramachandran plot, illustrating the distribution of amino acids within allowed and disallowed regions.

### 2.10. Linear and Conformational B-Cell Epitope Prediction

The Ellipro server (https://tools.iedb.org/ellipro/help/) was employed to detect both continuous and discontinuous B-cell epitopes within the refined 3D structure of the multiepitope constructs, utilizing default predictive parameters (0.5 and 6).

### 2.11. Vaccine–Receptor Docking and Interaction

Molecular docking involves the interaction of a ligand with its receptor, resulting in the creation of a stable complex [50]. Additionally, it can predict the binding strength between two molecules using specific scoring functions. For the docking simulations, we selected the TNF receptor and paired it with the optimized vaccine model. The 3D structure of the TNF receptor was created, refined, and validated using the servers outlined in Section 2.9. The ClusPro (https://cluspro.bu.edu/) server was utilized to evaluate the binding affinity between the multiepitope vaccines and the TNF receptor through molecular docking [51]. To scrutinize the interactions within the docked complex, DIMPLOT within LigPlot + v2.2 was utilized to analyze the interplay among residues of Chain-A (TNF receptor) and Chain-B (vaccine construct) [52]. The input was a PDB file depicting the vaccine–receptor complex. The output provided an in-depth representation of the intermolecular interactions, such as hydrogen bonds. Dotted lines in green and red visually depicted hydrogen bonds and salt bridges, respectively.

### 2.12. Normal Mode Analysis

To assess the macromolecular mobility and stability of the selected vaccine construct bound to the TNF receptor, we utilized the iMODS server (http://imods.chaconlab.org) [53]. We chose this particular server due to its superior efficiency and speed compared to other MD simulation methods available [54]. The iMODS server creates protein models that connect atoms using harmonic springs and employs normal mode analysis (NMA) to study the collective movement of proteins [55]. The server offers assessments like deformability, B-factors, eigenvalues, covariance maps, and elastic networks to analyze the flexibility, rigidity, and different types of motions (correlated, uncorrelated, and anticorrelated) within dynamic regions of the complex [56]. Deformability assesses a molecule's ability to alter the shape of its individual residues. B-factors, derived from NMA, gauge mobility in large molecules like proteins by multiplying the NMA mobility. Eigenvalues indicate motion rigidity and are linked to structural deformations—a lower value signifies easier deformation of the macromolecule. The covariance matrix demonstrates residue pair connections, influencing protein movements. The elastic network identifies pairs of atoms connected by springs.

### 2.13. Molecular Dynamics (MD) Simulation of Vaccine Constructs—Receptor Complexes

GROMACS 2020 and CHARMM36m force field conducted molecular dynamics (MD) simulations that model extensive biomolecular systems across time using efficient parallel algorithms [57]. The GROMACS software facilitated a 100 ns simulation of the docked complex involving the vaccine construct and TNF receptor. The GROMACS solvate tool was utilized to envelop the protein surface with a TIP3P water layer, spanning 10 Å. Sodium and chloride ions were introduced to neutralize the surface charges of the structure after the water layer addition. The system's atom count was verified, confirming a total of 407,960 atoms. To prevent undesired interactions between water and the complex, the system's energy was minimized using the steepest descent algorithm. Subsequently, the system underwent gradual heating from 0 to 300 K under constant volume for 200 ps, followed by equilibration at a consistent pressure. Throughout the 100-ns period, parameters including root–mean-square deviation (RMSD), root–mean-square fluctuation (RMSF), hydrogen bond count (H-bonds), radii of gyration (Rg), solvent accessible surface area (SASA), and ligand–receptor interaction energy were continuously computed.

### 2.14. Codon Optimization and In Silico Cloning

We employed a codon optimization strategy to enhance the expression of the recombinant protein. Given the degeneracy in the genetic code, where most amino acids can be encoded by multiple codons, optimizing codon usage becomes crucial. The Java Codon Adaptation Tool (JCat) server (http://www.prodoric.de/JCat) was utilized, specifically in the *Escherichia coli* (strain K12) codon system, to determine the codon adaptation index (CAI) values and GC contents, crucial indicators for assessing protein expression levels [58]. An optimal CAI value is 1.0, with scores above 0.8 considered favorable, while maintaining a GC content within the 30%–70% range. Translation and transcriptional efficiencies are adversely affected beyond these limits [59]. Subsequently, to confirm vaccine expression, the optimized sequence of the final vaccine construct, inclusive of restriction sites, was inserted into the *E. coli* plasmid vector pET−28 a (+) using SnapGene software (https://www.snapgene.com/free-trial/).

### 2.15. Immune Simulations

The C-ImmSim online server (https://kraken.iac.rm.cnr.it/C-IMMSIM/) was utilized to anticipate the immune response triggered by the vaccine. Employing the Celada-Seiden model, C-ImmSim portrays the immune system's dual-arm profile following exposure to the vaccine construct [60]. Simulations involved administering the vaccine without LPS and with three injections spaced at intervals of 1, 84, and 168. All other parameters were maintained at their default settings.

## 3. Results

### 3.1. The Sequences of the CD70, TNF-*α* Proteins, and CPP

The human CD70 and TNF-*α* reference sequences were sourced from NCBI and processed in FASTA format for subsequent analyses. Additionally, the CPP peptide sequences were obtained from a previous study for further use [24].

### 3.2. CTL Epitope Prediction

The CD70 protein sequence underwent analysis using the NetMHCpan 4.1 server to identify its most immunodominant regions. This involved assessing these regions against high-frequency HLA-I alleles. Notably, an inverse relationship between average rank scores and the binding affinity of epitopes to MHC-I alleles was observed. Higher binding affinity scores across multiple MHC-I alleles indicated superior CTL-stimulating epitopes. From the predicted epitopes, those displaying better binding affinity were chosen, outlined in Table 3. It is worth noting that all the selected epitopes were confirmed to be nonallergenic, nontoxic, and exhibited antigenicity (Table 3).

### 3.3. T Cell CD4^+^ Epitope Prediction

In the beginning, the exploration for potential CD70 epitopes in NetMHCIIpan-4.0 resulted in several sequences identified in humans. Following specific selection criteria, we refined the collection to two candidate CD70 epitopes for MHCII (Table 4). Considering their antigenic, allergenic, and toxic properties, the predicted CD70 epitopes were confirmed as nonallergenic, nontoxic, and exhibited antigenicity (Table 4).

### 3.4. MHC-I Processing and Immunogenicity of CTL Epitopes

Table 5 displays the proteasome score, TAP score, epitope identification score, and immunogenicity score for each chosen MHCI epitope. Notably, all epitopes listed in Table 5 exhibited high-quality proteasomal cleavage and efficient TAP transport.

### 3.5. Population Coverage

**Figure 1:**
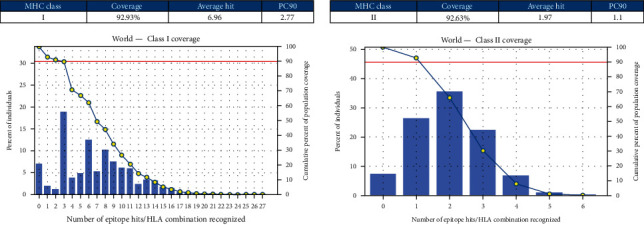
Population coverage analysis. Prediction of the population coverage for six potential human vaccine candidates with MHC Class I and II alleles around the world.

The top scoring epitopes and their respective population coverage percentage for human were indicated in Figure 1. The CD8^+^ and CD4^+^ T-cell epitopes showed a worldwide cumulative population coverage of 92%.

### 3.6. Prediction of Interferon-*γ* Inducing Epitope

Epitope prediction was carried out to identify sequences capable of inducing IFN-*γ* within the vaccine construct. IFN-*γ* plays a pivotal role in both innate and adaptive immune responses [61]. When triggered, T cells and NK (natural killer) cells produce IFN-*γ*, enhancing macrophage activation and antiviral mechanisms, while also augmenting antibody production [61]. Table 4 displays the cytokine secretion potential of the screened HTL epitopes in humans to induce IFN-*γ*. Positive SVM scores in IFN-*γ* induction were observed for all selected CD70 epitopes.

### 3.7. Peptide-Protein Flexible Docking

Upon assessing the interaction of MHC-peptide pairs, the top ten complexes were examined, and among them, model 0 displayed the most favorable lowest energy. Consequently, due to its superior interaction similarity scores between the CD70 epitopes and MHC-I and II molecules, model 0 was chosen and detailed in Tables 1 and 2.

### 3.8. Design of Multiepitope Constructs

In this investigation, we employed the SnapGene® 3.2.1 tool to design the final constructs, integrating the screened CTL and HTL epitopes sourced from the CD70 protein. Through this tool, we devised six structures encompassing the entire TNF protein alongside the CD70 and CPP epitopes. These constructs were arranged sequentially, connected by AAY linkers. Furthermore, the components of each vaccine construct were positioned adjacently, either at the N-terminal or C-terminal regions (CD70-CPP-TNF, CD70-TNF-CPP, CPP-CD70-TNF, CPP-TNF-CD70, TNF-CD70-CPP, and TNF-CPP-CD70). For visual reference, Figure S1 contains 2D representations of each construct.

### 3.9. The Physicochemical Parameters

The ProtParam server results were illustrated in Table 6. According to the data, the ideal molecular weight of the vaccine constructs falls within the range of 40–70 kDa [62]. Surprisingly, our developed vaccine constructs exhibit a fitting molecular weight of 45,530.11 Da for a vaccine construct, alongside a theoretical PI of 9.5, indicating the protein's basic characteristics. The protein instability index for the human constructs was calculated at 43.94. Moreover, the aliphatic index derived from the vaccine construction measured 91.22, while the GRAVY value stood at −0.166, suggesting a stable protein. The negative GRAVY value underscores the protein's hydrophilic nature [63]. Based on the solubility value of soluble *E. coli* protein, our vaccine constructs were anticipated to have low solubility [61]. Additionally, the constructs demonstrated antigenicity and were nonallergenic.

### 3.10. Tertiary Structure Prediction, Refinement, and Validation of 3D Structures

**Figure 2:**
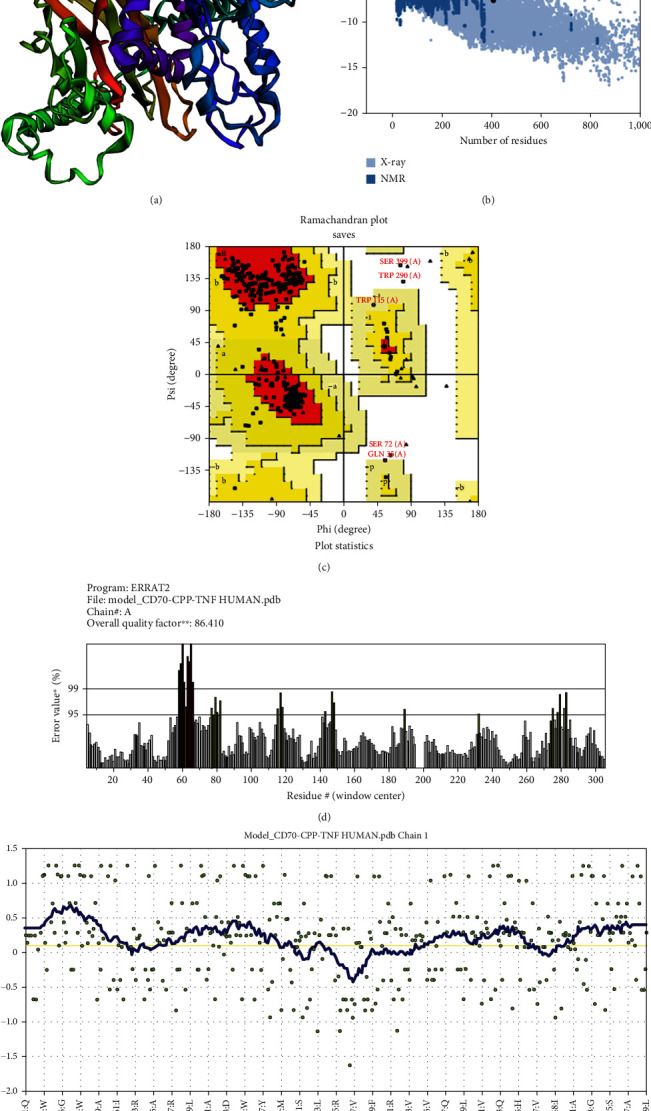
The outcomes from diverse structure validation tools affirmed the reliability and precision of the CD70^(epitopes)^−CPP-TNF^(Whole sequence)^ (*Homo sapiens*) vaccine construct. (a) The 3D model of the CD70^(epitopes)^−CPP-TNF^(Whole sequence)^ (*Homo sapiens*) vaccine construct was developed. (b) The ProSA-web *z*-score plot displayed a *Z*-score = −7.62. (c) The Ramachadran plot exhibited 89.5% of residues in the most favored regions. (d) The ERRAT overall quality factor exceeded 86%. (e) Postrefinement, the verify 3D indicated a score of 70.42% for the model.

The 3D models of the chimeric vaccine constructs generated by the Robetta server displayed favorable Ramachandran plot statistics. Across all constructs, over 82% of the residues was located within the favored and additionally allowed regions on the Ramachandran plot. The top 3D models were further enhanced using the GalaxyRefine 2 server, resulting in improved tertiary structure quality. Tools like ERRAT2, Verify3D, and PROCHECK hold high regard for evaluating the quality of protein structures [64–66]. Therefore, we performed an external quality assessment on the representative models using these tools. ERRAT2 calculates an overall quality factor representing the percentage of the protein structure with an error value below 95. This metric depends on the statistical distribution of pairwise atomic interactions, where a higher distribution of nonbonded atoms implies potential structural inaccuracies. The ERRAT score for the CD70-CPP-TNF vaccine stood at 86.41%, denoting good quality and validity (Figure 2). A score above 50 suggests a well-structured model [67]. Verify3D examines the compatibility between 3D models and their amino acid sequences (1D). Scores below 80% indicate that less than 80% of the amino acids in the structures achieved a score ≥0.1 in the 3D/1D profile. Specifically, the CD70-CPP-TNF model displayed 70.42% of residues with a 3D−1D score ≥0.1 (Figure 2). The vaccine constructs underwent validation via ProSA-web and Ramachandran plots postrefinement. The ProSA-web server yielded a Z-score of − 7.62 for the CD70-CPP-TNF chimeric vaccine (Figure 2), well within the acceptable range. In the Ramachandran plot, 89.5% of the residues were located in the most favored regions for this construct (Figure 2). These assessments collectively confirm the high quality of the final vaccines' 3D structures. Figures S2–S5 include the tertiary structures, ProSA-web *z*-score plots, Ramachandran plots, ERRAT, and verify 3D plots for the other chimeric constructs and TNF receptor.

### 3.11. Prediction of Structure-Based Epitopes on Vaccine Constructs

The epitope sequence should allow sufficient exposure and projection for B-cell receptors (BCRs) to bind effectively. Ellipro (http://tools.iedb.org/ellipro/) predicts both linear and discontinuous B-cell epitopes by utilizing the PI of a residue. This server was utilized to identify structural epitopes in the refined and validated 3D constructs. Tables 7 and 8 display the properties of the predicted linear and discontinuous B-cell epitopes for the CD70-CPP-TNF vaccine construct, respectively. Tables S1 and S2 present the predicted linear and conformational B-cell epitopes for other constructs. The scores and the number of epitopes obtained from the Ellipro server suggest that the predicted linear and discontinuous epitopes could potentially trigger B cell immune responses.

### 3.12. Protein–Protein Docking between TNF Receptor and Multiepitope Peptide Constructs

The ClusPro 2.0 software facilitated the simulation and analysis of potential interactions between the ligands of each vaccine construct and the TNF receptor. In total, seven models (CD70-CPP-TNF, CD70-TNF-CPP, CPP-CD70-TNF, CPP-TNF-CD70, TNF-CD70-CPP, TNF-CPP-CD70, and TNF-*α* whole sequence) were generated for each docking process. Models with the lowest energy scores were chosen. Notably, there exists an inverse relationship between energy levels and binding affinity; in simpler terms, lower energy levels indicate higher binding affinity between constructs and receptor in docked complexes. Intriguingly, when comparing the binding score of the TNF-*α* whole sequence with the TNF receptor, the vaccine constructs displayed higher docking scores with this receptor (Table 9). This suggests that the alignment and composition of the chimeric vaccines might enhance their interaction with the TNF receptor. To be more specific, the energy levels of docking between vaccine constructs and the TNF receptor could be overshadowed by the alignment of vaccine components. Interestingly, the CD70-CPP-TNF vaccine construct showed the minimum energy level of docking. The interacting amino acids between the receptor and ligand in the docked complexes were visualized using the DIMPLOT module in LigPlot+ as depicted in Figure S6.

### 3.13. Stability Prediction of the Vaccine-Receptor Complexes Molecular Dynamics (MD)

**Figure 3:**
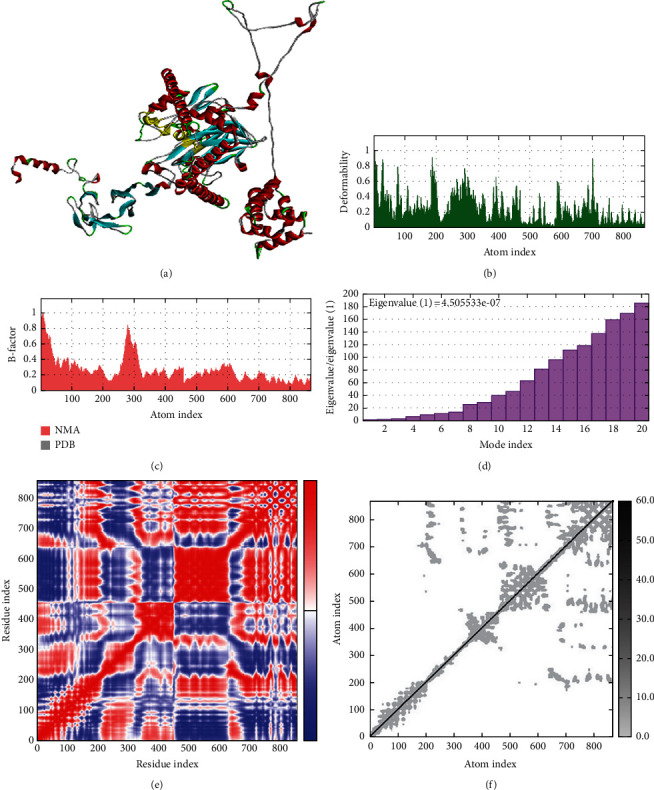
The iMOD server's normal mode analysis of the human CD70-CPP-TNF-TNF receptor complex with normal mode analysis (NMA) outputted the following plots. (a) The docked complex of the CD70-CPP-TNF-TNF receptor complex. Stability of the protein–protein complex was investigated through. (b) B-factor values, (c) deformability, (d) eigenvalue, (e) covariance map (red, white, and blue colors correspond to correlated, uncorrelated and anticorrelated motions), and (f) elastic network. The darker gray colors correspond to stiffer spring.

The iMOD server was employed to forecast the structural stability of the CD70-CPP-TNF-TNF-receptor complex, which displayed the highest docking scores. Illustrated in Figure 3(a) is the docked complex of CD70-CPP-TNF-TNF-receptor. In Figure 3(b), the peaks indicate regions of deformability within the ligand–receptor complex, while Figure 3(c) displays B factors as a gauge of protein mobility. Eigenvalues, representing the rigidity of the complexes, revealed that the CD70-CPP-TNF-TNF receptor (4.5 × 10^−7^) demanded considerable energy for complex deformation (Figure 3(d)). Generally, deformation becomes more difficult when the eigenvalue is high [55]. Furthermore, residue–residue contacts in the covariance matrix were visualized in red, while uncorrelated and anticorrelated motions were indicated in blue and white, respectively (Figure 3(e)). The elastic network model delineated atoms connected by springs, with stronger connections depicted by darker shades of gray (Figure 3(f)).

### 3.14. Molecular Dynamics Simulation

**Figure 4:**
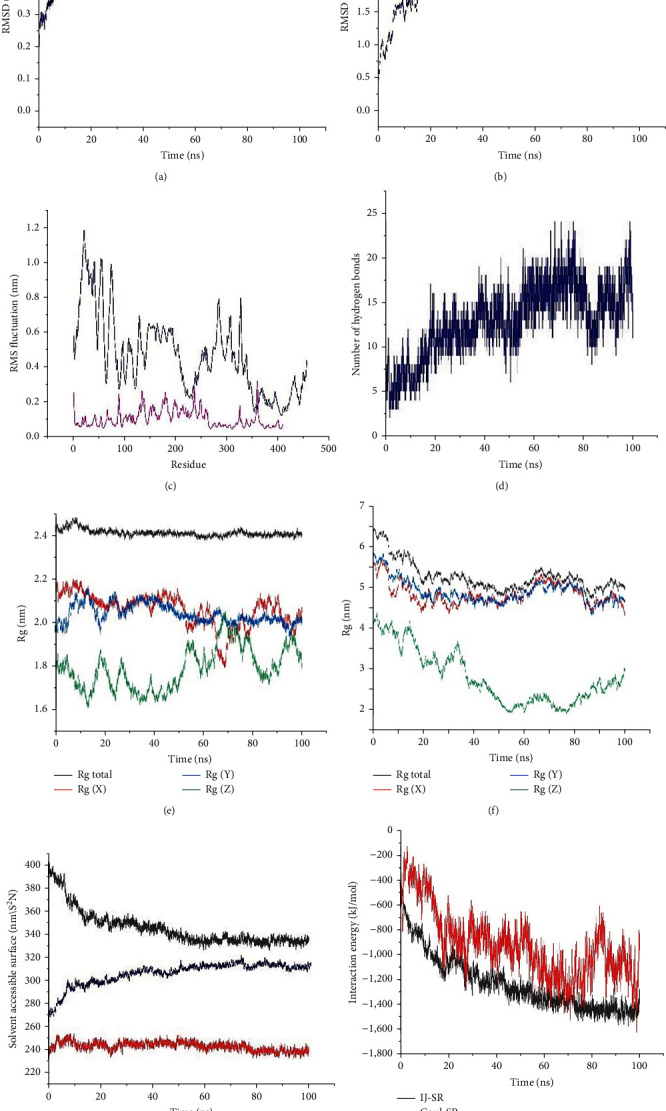
Based on the 100 ns MD simulations of the CD70-CPP-TNF–TNF receptor complex, the figure shows the: (a) root-mean-square deviation (RMSD) plot of vaccine. (b) RMSD plot of receptor. (c) Root-mean-square fluctuation (RMSF) plot of the ligand (blue line) and receptor (pink line). (d) Hydrogen bonds. (e) The radius of Gyration (Rg) plot of ligand. (f) The Rg plot of receptor. (g) Solvent accessible surface area (SASA) of the vaccine construct (red line), TNF receptor (blue line), and vaccine-TNF receptor complex (black line). (h) Interaction energy plot over the simulation timescale.

The MD simulation using GROMACS 2020 and CHARMM36m force field evaluated the stability of the CD70-CPP-TNF—TNF receptor complex through RMSD analysis. The vaccine's RMSD exhibited a progressive increase, stabilizing around 0.5 nm after 40 ns and remained steady throughout the simulation duration (Figure 4(a)). Initially, the receptor's RMSD surged sharply, reaching ∼0.25 nm by 60 ns, followed by minor fluctuations around this value until the simulation concluded (Figure 4(b)). Additionally, the RMSF, representing residual fluctuations, was assessed, measuring 0.47 nm for the receptor and 0.1 nm for the vaccine structure. The peaks on the graph indicate regions of high flexibility within the chains, as illustrated in Figure 4(c). Notably, the RMSF plot of the vaccine revealed a majority of residues displaying high flexibility (Figure 4(c)). Hydrogen bonds, pivotal for protein folding, stability, and flexibility, were observed within the range of 0.35 nm. A robust network of hydrogen bonds, ranging between 2 and 25 connections, was established between the chains (Figure 4(d)). The results suggest a progressive increase in the number of hydrogen bonds formed between the chains, affirming the stability and strong connection within the complex. Assessing the complex stability further, the Rg values were tracked across the MD trajectories. Rg measures the elastic stability of a cross-section. The average Rg value for the vaccine during the simulation was determined to be 5.26. Initially, the vaccine displayed a Rg of approximately 6.5 nm, which gradually decreased to 5 nm over the course of the simulation (Figure 4(e)). Conversely, the average Rg value for the TNF receptor during the simulation was found to be 2.41 nm. The TNF receptor consistently maintained low Rg values, remaining stable throughout the entire simulation period (Figure 4(f)).  Figure 4(g) depicted a notable rise in the TNF receptor's SASA over the simulation duration, while the SASA values of the vaccine remained constant, indicating the stability and compactness of its structure until the end of the simulation. The substantial SASA of the TNF receptor–vaccine complex indicates its stability within the solvent environment. Subsequently, we evaluated the nonbonded interaction energies—Lennard–Jones short-range (LJ-SR) and Coulombic short-range (Coul-SR) potential—between the TNF receptor and the vaccine (Figure 4(h)). Coul-SR accounts for the electrostatic interactions between the TNF receptor and the vaccine construct, while LJ-SR represents the van der Waals interactions between the two. Notably, the dominant interaction contributing to the stabilization of the TNF receptor and vaccine complex primarily arises from the electrostatic component, suggesting its critical role in binding.

### 3.15. Codon Optimization and In Silico Cloning

**Figure 5:**
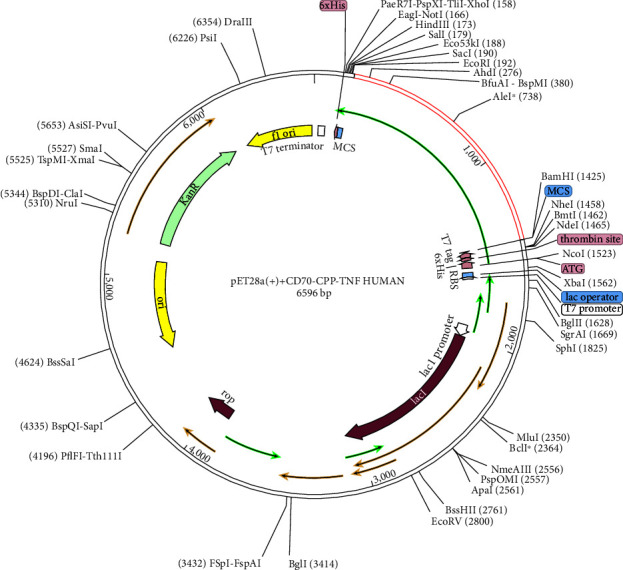
In silico cloning of human CD70-CPP-TNF vaccine construct into pET28A (+) vector. The red region denotes the 1233 bp cloned vaccine nucleic acid sequence.

We evaluated the cloning and expression efficiency of the vaccine. Additionally, we optimized the codon usage of the vaccine construct for *E. coli* strain K12. The JCat was employed for this purpose. The CAI value for the CD70-CPP-TNF construct was 0.960, with a mean GC proportion of 55.90%. These results suggest a high probability of successful expression of the vaccine in *E. coli*. After optimization, the codon sequences were incorporated into the pET28a (+) vector for cloning using SnapGene software. In Figure 5, the codon sequence of the final CD70-CPP-TNF vaccine is highlighted in red, representing the 1233 bp gene sequence generated by the JCat server. The pET28a (+) expression vector is illustrated in black. The codon sequence is inserted into the region between EcoRI and BamHI, resulting in a clone with a total length of 6596 bp.

### 3.16. Immune Simulation

**Figure 6:**
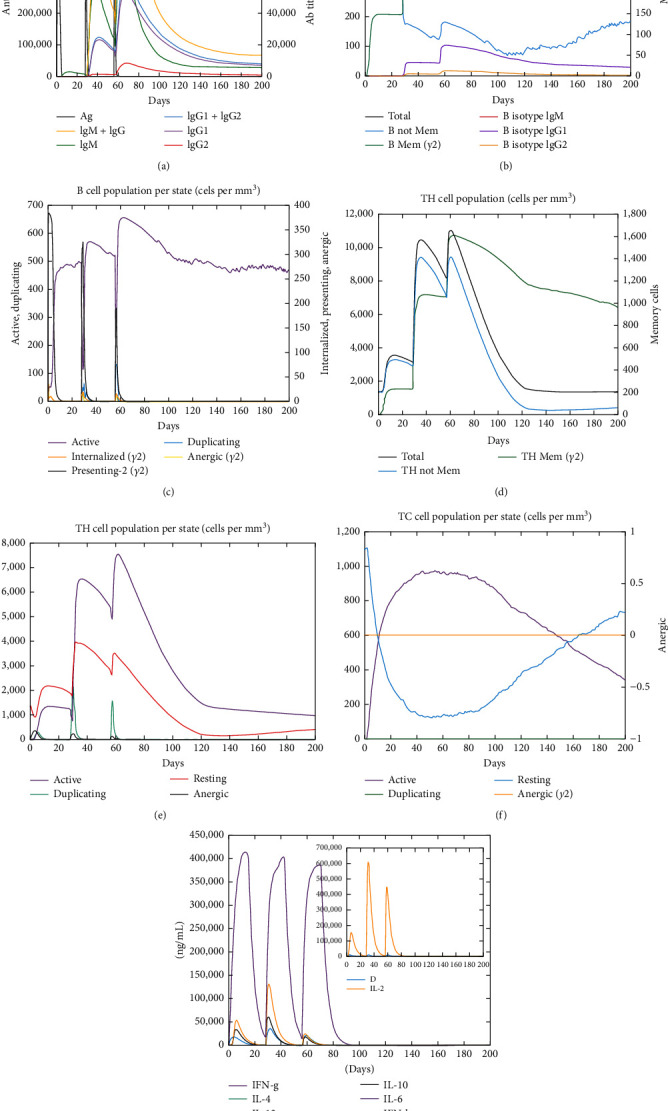
In silico simulation of immune response using CD70-CPP-TNF vaccine as an antigen after subsequent three injections. (a) Immunoglobulins and antigen levels, (b) B-cell population per state, (c) B-cell population, (d) T helper cell population per state, (e) T helper cell population, (f) T cytotoxic cell population per state, and (g) production of cytokines.

The immunogenic profile depicted in Figure 6 shows the response to the CD70-CPP-TNF multipeptide vaccine. Notably, three immunizations at 4-week intervals elicited a rapid and robust immune reaction. The initial CD70-CPP-TNF vaccine response exhibited heightened levels of IgM, followed by more robust secondary and tertiary responses characterized by elevated levels of IgM + IgG, IgG1 + IgG2, IgG1 antibodies, and a swift decline in antigen concentration (Figure 6(a)). Substantial B-cell activation, particularly in B isotypes IgM and IgG1, was observed, along with noticeable memory cell development (Figure 6(b)). Furthermore, both TH and TC populations showed a heightened presence, along with memory cell proliferation (Figure 6(c)–6(f)). The vaccine demonstrated the ability to induce both IFN-*γ* and IL-2 responses, with a satisfactory Simpson index (*D*) (Figure 6(g)), indicative of diversity in the response.

## 4. Discussion

The rise of RCC poses a significant global public health threat, leading to heightened rates of mortality and morbidity. Researchers have been motivated to develop an efficient treatment in response to this situation. Vaccination stands as a widely utilized method to bolster the host's immune response during disease scenarios [68]. Immunoinformatic offers advantages in vaccine design by allowing rapid screening of potential epitopes, predicting immunogenicity, and aiding in the selection of the most effective candidates for vaccine development [69, 70]. Chimeric or multivalent antigens, which link potentially immunogenic molecules into a unified structure, present an appealing approach [71]. For decades, researchers have dedicated their efforts to discovering a potent antigen and formulation for an RCC vaccine, resulting in the identification of promising antigens and a shared perspective on the significance of vaccination in tackling the disease [72].

A recent study by Panowski et al. [73], demonstrated the development of CAR-T cells directed at CD70 single-chain antibodies. These anti-CD70 CAR-T cells demonstrated robust antitumor effects against RCC. This discovery underscores the potential of CD70 CAR-T cells in RCC treatment, leading to an ongoing Phase I clinical trial assessing the efficacy of CD70-CAR-T cells in metastatic RCC. Consequently, our main objective in this current study was to design a multiepitope vaccine encompassing T and B-cell epitopes derived from CD70 using immunoinformatics analysis. Extensive evidence supports the ability of antigenic epitopes to effectively trigger specific immune responses targeting the complete antigen [74]. We employed NetMHCpan 4.0 and NetMHCIIpan 4.1 servers to uncover numerous potential epitopes within the CD70 protein. Epitopes were filtered based on their antigenic characteristics. Notably, none of the selected epitopes have been found in any studies. The chosen epitopes showed superior scores in MHC binding and extensive coverage across various MHCI and MHCII subtypes. Efficient antigen processing and optimal presentation to the immune system are vital for a strong CD8^+^ T cell response. Hence, we assessed T cell antigen processing using the NETCTLpan1.1 server in this study. Each MHCI epitope exhibits a high-quality proteasomal cleavage and efficient TAP transport. Higher processing scores denote enhanced antigen processing outcomes [75, 76]. Given IFN-*γ*'s pivotal role in acquired and innate immunity against tumors [77], we proceeded to assess the IFN-*γ* inducing HTL epitopes. All chosen epitopes were proficient in inducing IFN-*γ*.

Due to the importance of HLA specificity in T-cell epitope selection [77], we employed molecular docking simulations to establish the connections between these epitopes and their respective HLA alleles. The analyses revealed robust binding in the epitope-MHC complexes, evident from their higher global energy scores.

Combining individual peptides into chimeric formations diminishes the risk of degradation by endo- or exopeptidases and augments their antigenicity, considering that individual peptides tend to have low immune-stimulating properties [78]. In the pursuit of heightened antigenicity, the highest ranked epitopes were united using a suitable linker, culminating in the structure of the chimeric vaccine. Additionally, the final vaccine structure incorporated TNF-*α* adjuvant and CPP peptide to bolster the elicited immune response [79]. In a multiepitope vaccination strategy, each epitope functions as an individual immunological unit. Consequently, careful scrutiny of their arrangement and placement within the designed constructs is imperative [80]. Six multiepitope vaccines—CD70-CPP-TNF, CD70-TNF-CPP, CPP-CD70-TNF, CPP-TNF-CD70, TNF-CD70-CPP, and TNF-CPP-CD70, were developed. These constructs incorporated seven MHC class-I and two MHC class-II epitopes, TNF-*α* as an adjuvant, and the CPP peptide. The TNF-*α* whole sequence and CPP were strategically positioned at the C-terminal and N-terminal regions of the immunogenic CD70 epitopes, connected by the AAY linker.

Given the potential variance in the immunogenicity of individual epitopes when combined, it was crucial to assess the antigenicity of the designed constructs. Results from VaxiJen v2 affirmed the antigenicity of all constructs. A proficient vaccine should not only evoke strong immune responses but also maintain favorable physicochemical and structural properties during production. Evaluation of the construct's physicochemical characteristics indicated their compliance with acceptable standards. Additionally, the assessment affirmed that the constructs posed no allergenicity concerns, ensuring the vaccine's safety profile.

Ensuring the quality of the tertiary structure in the vaccine construct is crucial as it significantly influences the presentation of peptides that trigger immune responses [81]. The Robetta server was used for initial 3D modeling of the constructs, followed by refinement through Galaxy Refine, resulting in validated output models. The refinement notably elevated the tertiary structure of the vaccine to the desired standard. The ElliPro server predicted several linear and conformational B cell epitopes, indicating the constructs' potential to trigger immune responses encompassing both cellular and humoral aspects.

It is crucial to highlight that for the vaccine to exert its adjuvant effect, it must possess the capability to bind to the TNF receptor. The activation of innate immune cells through the TNF receptor is integral to the stimulation of cells in the adaptive immune system. In this context, the docking analysis of the TNF receptor was examined. Our findings reveal novel peptide vaccines that interact effectively with TNF receptors, signifying satisfactory vaccine uptake by antigen-presenting cells (APCs). It is worth noting that the docking energy between the vaccine constructs and receptors might be impacted by how the vaccine components align. Specifically, TNF-*α* linked to CPP or CD70 epitopes demonstrated stronger docking with the TNF receptor compared to TNF-*α* alone. In general, CD70-CPP-TNF exhibited encouraging outcomes regarding its docking with the TNF receptor.

Fascinatingly, studies have demonstrated that CPPs effectively transport proteins and peptides across various cell types [82]. Therefore, incorporating CPPs into our vaccine design significantly enhanced the uptake of the vaccines by APCs.

The NMA data indicated that the docked proteins exhibit minimal deformability, suggesting a robust and stable binding capability of the vaccine-immune cell receptor complex. The anticipated eigenvalue of the vaccine-TNF receptor complex signifies its stability. Both the covariance and elastic network diagrams portray regions with correlated motions among amino acids and inflexible segments (signified by stiffer springs), respectively. The existence of these elements confirms the overall stability of the vaccine–receptor complex [83].

To assess the stability of the CD70-CPP-TNF—TNF receptor complex, molecular dynamics simulations were conducted. The RMSD results indicate the overall stability of the complex. Peaks in the graph represent highly flexible regions, while moderate fluctuations indicate relatively rigid areas. Based on Figure 4(c), there is evident fluctuation noticed within residues 1–170, which corresponds to the linker regions in the multiepitope vaccine structure. Following that, our focus shifted to examining the quantity of hydrogen bonds within the complex of the TNF receptor and the vaccine construct, which exhibited a gradual increase over the course of the simulations. This analysis further suggests the establishment of a stable interaction between the TNF receptor and the vaccine structure. Moreover, the Rg graphs for both the vaccine and receptor proteins demonstrate that the TNF receptor displays a lower average Rg fluctuation value compared to the vaccine. The higher SASA value observed for the vaccine–receptor complex indicates its stability within the solvent. Additionally, the energy assessment revealed that the electrostatic interaction between the TNF receptor and vaccine contributes significantly to stabilizing the complex. In the process of developing a multiepitope vaccine, a crucial stage involves effective cloning and expression within a compatible vector [84]. Given the redundancy in the genetic code, necessitating multiple codons to encode one amino acid [59], we carried out codon optimization and simulated cloning. This yielded enhanced expression levels and efficient translation of the vaccine when using pET-28a (+).

Since, the vaccine comprises CTL and HTL epitopes, it has the potential to activate corresponding immune cells within the host. This activation could subsequently initiate signaling pathways that stimulate other immune cells [79]. The counts of helper and cytotoxic T cells notably increased after the initial dose and continued to rise following subsequent doses. B cells also mirrored this trend: the primary response showed increased IgM levels, while the secondary and tertiary responses surpassed the primary one, displaying higher levels of IgM + IgG, IgG1 + IgG2, IgG1 antibodies, and a swift decrease in antigen concentration. The simulation using the construct prompted the production of IFN-*γ* and IL-2, supported by a suitable Simpson index (*D*) reflecting diversity. Moreover, the CD70-CPP-TNF construct triggered a minimal amount of IL-10, suggesting its potential to stimulate a TH1-mediated immune response, crucial for immunity against RCC. In sum, the designed vaccine holds significant promise from an immunological standpoint. Its immunogenicity stems from the selection of epitopes with high binding affinities to MHC class I and II alleles, crucial for robust T cell responses. The inclusion of CPPs and TNF-*α* sequences in the vaccine enhances antigenicity and aids in immune cell uptake, triggering a cascade of immune reactions. Moreover, the predicted B-cell epitopes suggest elicitation of a potent humoral response, crucial for long-term immunity.

## 5. Conclusion

In this research, we utilized computer-based techniques to create a multiepitope vaccination for CD70 as prophylaxis to human RCC. To the best of our knowledge, this study is the first to design potential vaccines against CD70-overexpressing RCC. Our vaccine design displayed considerable immune response. Nevertheless, additional examinations both *in vitro* and *in vivo* are necessary to confirm its actual capacity to trigger an effective immune response concerning RCC. However, translating *in silico* findings into experimental contexts comes with limitations and potential challenges. The reliance on algorithms for predictive accuracy and immunogenicity might not fully encompass the intricacies of the immune system. Also, biological variability, encompassing genetic, environmental, and health-related factors, could impact vaccine efficacy across different individuals. In addition, determining the long-term effectiveness and durability of the immune response induced by the designed vaccine poses challenges. *In silico* models may not precisely predict the longevity and robustness of the immune memory. Further, the practical manufacturing and scalability of a vaccine based on *in silico* results might face unexpected hurdles like production scalability, stability, and cost-effectiveness. Overcoming these challenges demands a multidisciplinary approach involving computational biologists, immunologists, clinicians, regulatory bodies, and industry partners [85].

## Figures and Tables

**Table 1:** Interaction similarity scores of the selected CTL epitopes in CD70 protein.

Protein name	Epitope	HLA-A01:01 (PDB ID:4NQV)	HLA-A02:01 (PDB ID:4UQ3)	HLA-A03:01 (PDB ID:3RL2)	HLA-A24:02 (PDB ID:5HGA)	HLA-A11:01 (PDB ID:1 × 7Q)	HLA-B07:02 (PDB ID:5EO1)	HLA-B08:01 (PDB ID:3SPV)	HLA-B27:05 (PDB ID:1OGT)	HLA-B35:01 (PDB ID:3LKN)	HLA-B51:01 (PDB ID:1E27)	Average of interaction similarity score ^*∗*^
CD70 (*Homo sapiens*)	CD70 (43–55)	211	245.5	228	226.5	229	240	229.5	235.5	263.5	260	236.85
	CD70 (70–83)	208.7	265.3	221.2	242.9	218.5	216.3	229	215.9	219.9	218.1	225.58
	CD70 (72–86)	208.7	265.5	221.2	243.6	218.5	218.4	225.8	216.1	225.2	215.5	225.85
	CD70 (99–112)	174.8	209.6	190.1	248	187.4	207	230.3	190.6	191.7	194	202.35
	CD70 (118–132)	164.4	212.8	117.4	188.9	175.1	184.6	178.6	173.3	188.9	253	183.7
	CD70 (134–147)	162	216	180.8	198	178	196.7	187.9	194.2	236.9	207.6	195.81
	CD70 (138–147)	144.4	183.5	163.5	196	160.3	170.7	157.9	173.6	235	190	177.49

^*∗*^Higher rate shows better quality of peptide-MHC interactions.

**Table 2:** Interaction similarity scores of the selected HTL epitopes in CD70.

Protein name	Epitope	DRB1:0101 (PDB ID:4AH2)	DRB1:0301 (PDB ID:2Q6W)	DRB1:0401 (PDB ID:5LAX)	DRB1:1101 (PDB ID:6CPL)	DRB1:1501 (PDB ID:5V4M)	DRB5:0101 (PDB ID:1FV)	Average
CD70 (*Homo sapiens*)	CD70 (54–72)	122.22	130.6	130.66	132.1	129.6	125.8	128.49
	CD70 (69–84)	122.4	138.8	116.8	113.6	115.3	113.6	120.08

^*∗*^Higher rate shows better quality of peptide-MHC interactions.

**Table 3:** Final selected CTL epitopes from CD70 protein for constructing multiepitope vaccine witht their corresponding MHC Class I alleles and immunogenic traits.

Protein name	Position	Epitope sequence	No. of allels	Allels	NetMHCpan average rank scores ^*∗*^	Allergenicity	Antigenicity ^*∗∗*^	Toxicity
CD70	43–55	QAQQQLPLESLGW	9	HLA-B ^*∗*^58:01 HLA-B ^*∗*^39:01 HLA-B ^*∗*^15:01 HLA-B ^*∗*^13:01 HLA-B ^*∗*^40:01 HLA-B ^*∗*^08:01 HLA-A ^*∗*^32:01 HLA-A ^*∗*^24:02 HLA-B ^*∗*^35:01	1.027	Nonallergen	0.6649	Non
	70–83	DPRLYWQGGPALGR	10	HLA-B ^*∗*^07:02 HLA-A ^*∗*^24:02 HLA-A ^*∗*^02:01 HLA-B ^*∗*^15:01 HLA-A ^*∗*^32:01 HLA-B ^*∗*^13:01 HLA-A ^*∗*^03:01 HLA-A ^*∗*^11:01 HLA-B ^*∗*^39:01 HLA-A ^*∗*^33:01	0.683	Nonallergen	0.6572	Non
	72–86	RLYWQGGPALGRSFL	11	HLA-A ^*∗*^02:01 HLA-A ^*∗*^24:02 HLA-B ^*∗*^07:02 HLA-B ^*∗*^15:01 HLA-A ^*∗*^32:01 HLA-B ^*∗*^13:01 HLA-A ^*∗*^03:01 HLA-A ^*∗*^11:01 HLA-B ^*∗*^39:01 HLA-A ^*∗*^33:01 HLA-B ^*∗*^35:01	0.664	Nonallergen	0.5323	Non
CD70 (*Homo sapiens*)	99–112	HRDGIYMVHIQVTL	8	HLA-B ^*∗*^39:01 HLA-B ^*∗*^27:05 HLA-A ^*∗*^02:01 HLA-A ^*∗*^24:02 HLA-B ^*∗*^08:01 HLA-B ^*∗*^15:01 HLA-A ^*∗*^32:01 HLA-B ^*∗*^13:01	0.778	Nonallergen	0.9996	Non
	118–132	TTASRHHPTTLAVGI	10	HLA-B ^*∗*^39:01 HLA-B ^*∗*^07:02 HLA-B ^*∗*^08:01 HLA-B ^*∗*^58:01 HLA-B ^*∗*^15:01 HLA-A ^*∗*^30:01 HLA-A ^*∗*^32:01 HLA-B ^*∗*^13:01 HLA-B ^*∗*^27:05 HLA-B ^*∗*^35:01	0.886	Nonallergen	0.9327	Non
	134–147	SPASRSISLLRLSF	16	HLA-B ^*∗*^07:02 HLA-B ^*∗*^08:01 HLA-B ^*∗*^39:01 HLA-B ^*∗*^35:01 HLA-A ^*∗*^33:01 HLA-A ^*∗*^68:01 HLA-A ^*∗*^03:01 HLA-A ^*∗*^11:01 HLA-A ^*∗*^30:01 HLA-B ^*∗*^27:05 HLA-B ^*∗*^58:01 HLA-B ^*∗*^15:01 HLA-A ^*∗*^32:01 HLA-A ^*∗*^24:02 HLA-A ^*∗*^26:01 HLA-B ^*∗*^13:01	0.605	Nonallergen	1.1247	Non
	138–147	RSISLLRLSF	10	HLA-B ^*∗*^58:01 HLA-A ^*∗*^30:01 HLA-B ^*∗*^15:01 HLA-A ^*∗*^32:01 HLA-A ^*∗*^24:02 HLA-A ^*∗*^26:01 HLA-B ^*∗*^07:02 HLA-B ^*∗*^08:01 HLA-B ^*∗*^13:01 HLA-B ^*∗*^35:01	0.872	Nonallergen	1.4089	Non

^*∗*^Lower rates show better binding affinity.  ^*∗∗*^Higher rate shows high degree of peptide antigenicity.

**Table 4:** Final selected HTL epitopes from CD70 protein for constructing multiepitope vaccine with their corresponding MHC Class II alleles and immunogenic traits.

Protein name	Position	Epitope sequence	No. of allels	Allels	NetMHCIIpan average rank scores ^*∗*^	Allergenicity	Antigenicity ^*∗∗*^	Toxicity	IFN-*γ* –induction
CD70 (*Homo sapiens*)	54–72	GWDVAELQLNHTGPQQDPR	6	DRB4_0101 DRB1_1302 DRB3_0202 DRB1_0401 DRB1_0802 HLA-DQA10301-DQB10302	2.71	Nonallergen	1.0262	Non	Inducer
	69–84	QDPRLYWQGGPALGRS	6	DRB1_0101 DRB1_0901 DRB1_1501 HLA-DQA10501-DQB10201 HLA-DQA10501-DQB10301 DRB1_1601	2.45	Nonallergen	0.5512	Non	Inducer

^*∗*^Lower rates show better binding affinity.  ^*∗∗*^Higher rate shows high degree of peptide antigenicity.

**Table 5:** MHC-I processing prediction and immunogenicity scores of CD70 CTL epitopes.

Protein name	Position	Epitope sequence	TAP transport efficiency score ^*∗*^	Proteasomal C terminal cleavage score ^*∗∗*^	Epitope identification score ^*∗∗∗*^	immunogenicity scores ^*∗∗∗∗*^
CD70 (*Homo sapiens*)	43–55	QAQQQLPLESLGW	1.097	0.812	0.8	−0.34715
	70–83	DPRLYWQGGPALGR	1.758	0.629	0.55	0.20741
	72–86	RLYWQGGPALGRSFL	2.002	0.632	0.5	0.20189
	99–112	HRDGIYMVHIQVTL	1.202	0.976	0.33	0.14608
	118–132	TTASRHHPTTLAVGI	1.3	0.939	0.8	0.1051
	134–147	SPASRSISLLRLSF	2.39	0.871	0.431	0.37487
	138–147	RSISLLRLSF	2.98	0.883	0.514	0.20217

^*∗*^Higher score shows better quality of TAP transport efficiency,  ^*∗∗*^higher score shows better quality of proteasomal cleavage,  ^*∗∗∗*^higher rates show better quality of epitope identification,  ^*∗∗∗*^higher score indicates a greater probability of eliciting an immune response.

**Table 6:** Physicochemical characteristics of the constructed vaccine.

Construct	Molecular weight (kDa)	Theoretical PI	Positive charge residue	Negative charge residue	Solubility	Instability index of constructs	Allergenicity	Antigenicity
Human CD70-CPP-TNF	45530.11	9.50	42	30	0.184	43.94	Nonallergen	0.644

**Table 7:** Predicted linear epitopes of CD70 (*Homo sapiens*) peptide vaccine constructs.

Construct	No.	Chain	Start	End	Peptide	Number of residues	Score
CD70-CPP-TNF (*Homo sapiens*)	1	A	180	247	ESMIRDVELAEEALPKKTGGPQGSRRCLFLSLFSFLIVAGATTLFCLLHFGVIGPQREEFPRDLSLIS	68	0.818
	2	A	335	341	ITVSYQT	7	0.797
	3	A	354	365	QRETPEGAEAKP	12	0.759
	4	A	114	166	AAYGWDVAELQLNHTGPQQDPRAAYQDPRLYWQGGPALGRSAAYRQIKIWFQN	53	0.711
	5	A	86	93	YSPASRSI	8	0.707
	6	A	66	76	AAYTTASRHHP	11	0.652
	7	A	377	383	QLEKGDR	7	0.643
	8	A	301	305	VVPSE	5	0.638
	9	A	254	263	RSSSRTPSDK	10	0.592
	10	A	52	55	HRDG	4	0.578
	11	A	290	298	ANGVELRDN	9	0.533
	12	A	319	326	QGCPSTHV	8	0.52
	13	A	1	11	QAQQQLPLESL	11	0.517

**Table 8:** Predicted Discontinuous epitopes of CD70 ligand (*Homo sapiens*) peptide vaccine constructs.

Construct	No.	Residues	Number of residues	Score
CD70-CPP-TNF (*Homo sapiens*)	1	A:E238, A:F239, A:P240, A:R241, A:D242, A:L243, A:S244, A:L245, A:I246, A:S247, A:Q251	11	0.788
	2	A:Y176, A:M177, A:E180, A:S181, A:I183, A:R184, A:D185, A:V186, A:E187, A:L188, A:A189, A:E190, A:E191, A:A192, A:L193, A:P194, A:K195, A:K196, A:T197, A:G198, A:G199, A:P200, A:Q201, A:G202, A:S203, A:R204, A:R205, A:C206, A:L207, A:F208, A:L209, A:S210, A:L211, A:F212, A:S213, A:F214, A:L215, A:I216, A:V217, A:A218, A:G219, A:A220, A:T221, A:T222, A:L223, A:F224, A:C225, A:L226, A:L227, A:H228, A:F229, A:G230, A:V231, A:I232, A:G233, A:P234, A:Q235, A:N271, A:P272, A:Q273, A:A274, A:E275, A:N282, A:R283, A:Q319, A:G320, A:C321, A:P322, A:S323, A:T324, A:H325, A:C353, A:Q354, A:R355, A:E356, A:T357, A:P358, A:E359, A:G360, A:A361, A:E362, A:A363, A:K364, A:P365	84	0.749
	3	A:T64, A:A66, A:A67, A:Y68, A:T69, A:T70, A:A71, A:S72, A:R73, A:H74, A:H75, A:P76, A:T77, A:S87, A:P88, A:A89, A:S90, A:R91, A:S92, A:I93, A:S99, A:Y103, A:R104, A:S105, A:I106, A:S107, A:R110, A:A114, A:A115, A:G117, A:W118, A:D119, A:V120, A:A121, A:E122, A:L123, A:Q124, A:L125, A:N126, A:H127, A:T128, A:G129, A:P130, A:Q131, A:Q132, A:D133, A:P134, A:R135, A:A136, A:A137, A:Q139, A:D140, A:P141, A:R142, A:L143, A:Y144, A:W145, A:Q146, A:G147, A:G148, A:P149, A:A150, A:L151, A:G152, A:R153, A:S154, A:A155, A:A156, A:Y157, A:R158, A:Q159, A:I160, A:K161, A:I162, A:W163, A:Q165, A:N166, A:M169	78	0.697
	4	A:R254, A:S255, A:S257, A:R258, A:T259, A:P260, A:S261, A:D262, A:K263, A:P264, A:L288, A:A290, A:N291, A:G292, A:V293, A:E294, A:R296, A:D297, A:N298, A:V301, A:V302, A:P303, A:S304, A:E305, A:I335, A:T336, A:V337, A:S338, A:Y339, A:Q377, A:E379, A:K380, A:G381, A:D382, A:R383	35	0.624
	5	A:Q1, A:A2, A:Q3	3	0.614

**Table 9:** Protein–Protein docking results (lowest energy in the best model) between final vaccine constructs and TNF receptor.

Construct	TNF receptor (kcal/mol)
CD70-CPP-TNF	−1570.1
CD70-TNF-CPP	−1487.6
CPP-CD70-TNF	−1499.0
CPP-TNF-CD70	−1505.7
TNF-CD70-CPP	−1397.7
TNF-CPP-CD70	−1453.4
TNF whole	−1271.1

## Data Availability

The data used to support the findings of this study are included within the article or within the supplementary information file.

## Supplementary Materials

Supplementary Materials Figure S1: that shows 2D structure of vaccine constructs, Figures S2–S5: that correspond to the refined and validated structure of vaccines and tumor necrosis factor receptor (TNFR), and Figure S6: representing Ligplot analysis showing the hydrogen bonding and hydrophobic interactions between TNFR and vaccine constructs. This file also includes Tables S1 and S2: which, respectively, contain predicted linear and discontinues epitopes of CD70 peptide vaccine constructs.

## References

[1] Motzer R. J., Mazumdar M., Bacik J., Russo P., Berg W. J., Metz E. M. (2000). Effect of cytokine therapy on survival for patients with advanced renal cell carcinoma. *Journal of Clinical Oncology*.

[2] Junker K., Hindermann W., von Eggeling F., Diegmann J., Haessler K., Schubert J. (2005). CD70: a new tumor specific biomarker for renal cell carcinoma. *Journal of Urology*.

[3] Deleuze A., Saout J., Dugay F. (2020). Immunotherapy in renal cell carcinoma: the future is now. *International Journal of Molecular Sciences*.

[4] Uemura H., Fujimoto K., Tanaka M. (2006). A phase I trial of vaccination of CA9-derived peptides for HLA-A24-positive patients with cytokine-refractory metastatic renal cell carcinoma. *Clinical Cancer Research*.

[5] Bleumer I., Tiemessen D. M., Oosterwijk-Wakka J. C. (2007). Preliminary analysis of patients with progressive renal cell carcinoma vaccinated with CA9-peptide-pulsed mature dendritic cells. *Journal of Immunotherapy*.

[6] Iiyama T., Udaka K., Takeda S. (2007). WT1 (Wilms’ tumor 1) peptide immunotherapy for renal cell carcinoma. *Microbiology and Immunology*.

[7] Patel P. M., Sim S., O’Donnell D. O. (2008). An evaluation of a preparation of mycobacterium vaccae (SRL172) as an immunotherapeutic agent in renal cancer. *European Journal of Cancer*.

[8] Rahma O. E., Ashtar E., Ibrahim R. (2010). A pilot clinical trial testing mutant von Hippel–Lindau peptide as a novel immune therapy in metastatic renal cell carcinoma. *Journal of Translational Medicine*.

[9] Kim D. W., Krishnamurthy V., Bines S. D., Kaufman H. L. (2010). TroVax, a recombinant modified vaccinia Ankara virus encoding 5T4: lessons learned and future development. *Human Vaccines*.

[10] Amato R. J., Hawkins R. E., Kaufman H. L. (2010). Vaccination of metastatic renal cancer patients with MVA-5T4: a randomized, double-blind, placebo-controlled phase III study. *Clinical Cancer Research*.

[11] Stephens A. J., Burgess-Brown N. A., Jiang S. (2021). Beyond just peptide antigens: the complex world of peptide-based cancer vaccines. *Frontiers in Immunology*.

[12] Ernstoff M. S., Crocenzi T. S., Seigne J. D. (2007). Developing a rational tumor vaccine therapy for renal cell carcinoma: immune Yin and Yang. *Clinical Cancer Research*.

[13] Walter S., Weinschenk T., Stenzl A. (2012). Multipeptide immune response to cancer vaccine IMA901 after single-dose cyclophosphamide associates with longer patient survival. *Nature Medicine*.

[14] Rini B. I., Stenzl A., Zdrojowy R. (2016). IMA901, a multipeptide cancer vaccine, plus sunitinib versus sunitinib alone, as first-line therapy for advanced or metastatic renal cell carcinoma (IMPRINT): a multicentre, open-label, randomised, controlled, phase 3 trial. *The Lancet Oncology*.

[15] Kirner A., Mayer-Mokler A., Reinhardt C. (2014). IMA901: a multi-peptide cancer vaccine for treatment of renal cell cancer. *Human Vaccines & Immunotherapeutics*.

[16] Lens S. M. A., Drillenburg P., Den Drijver B. F. A. (1999). Aberrant expression and reverse signalling of CD70 on malignant B cells. *British Journal of Haematology*.

[17] Diegmann J., Junker K., Gerstmayer B. (2005). Identification of CD70 as a diagnostic biomarker for clear cell renal cell carcinoma by gene expression profiling, real-time RT–PCR and immunohistochemistry. *European Journal of Cancer*.

[18] Law C.-L., Gordon K. A., Toki B. E. (2006). Lymphocyte activation antigen CD70 expressed by renal cell carcinoma is a potential therapeutic target for anti-CD70 antibody-drug conjugates. *Cancer Research*.

[19] Ryan M. C., Kostner H., Gordon K. A. (2010). Targeting pancreatic and ovarian carcinomas using the auristatin-based anti-CD70 antibody-drug conjugate SGN-75. *British Journal of Cancer*.

[20] Kim T. J., Lee Y. H., Koo K. C. (2022). Current and future perspectives on CAR-T cell therapy for renal cell carcinoma: a comprehensive review. *Investigative and Clinical Urology*.

[21] Adam P. J., Terrett J. A., Steers G. (2006). CD70 (TNFSF7) is expressed at high prevalence in renal cell carcinomas and is rapidly internalised on antibody binding. *British Journal of Cancer*.

[22] Albinger N., Hartmann J., Ullrich E. (2021). Current status and perspective of CAR-T and CAR-NK cell therapy trials in Germany. *Gene Therapy*.

[23] Kim I.-H., Lee H. J. (2022). The frontline immunotherapy-based treatment of advanced clear cell renal cell carcinoma: current evidence and clinical perspective. *Biomedicines*.

[24] Backlund C. M., Holden R. L., Moynihan K. D. (2022). Cell-penetrating peptides enhance peptide vaccine accumulation and persistence in lymph nodes to drive immunogenicity. *Proceedings of the National Academy of Sciences*.

[25] Kayamuro H., Abe Y., Yoshioka Y. (2009). The use of a mutant TNF-*α* as a vaccine adjuvant for the induction of mucosal immune responses. *Biomaterials*.

[26] Dey J., Mahapatra S. R., Singh P. K., Prabhuswamimath S. C., Misra N., Suar M. (2023). Designing of multi-epitope peptide vaccine against acinetobacter baumannii through combined immunoinformatics and protein interaction–based approaches. *Immunologic Research*.

[27] Boyaka P. N., Marinaro M., Jackson R. J. (1999). IL-12 is an effective adjuvant for induction of mucosal immunity. *The Journal of Immunology*.

[28] Staats H. F., Bradney C. P., Gwinn W. M. (2001). Cytokine requirements for induction of systemic and mucosal CTL after nasal immunization. *The Journal of Immunology*.

[29] Wajant H., Pfizenmaier K., Scheurich P. (2003). Tumor necrosis factor signaling. *Cell Death & Differentiation*.

[30] Brunner C., Seiderer J., Schlamp A. (2000). Enhanced dendritic cell maturation by TNF-*α* or cytidine-phosphate-guanosine DNA drives T cell activation in vitro and therapeutic anti-tumor immune responses in vivo. *The Journal of Immunology*.

[31] Chen Z., Huang H., Chang T. (2002). Enhanced HER-2/neu-specific antitumor immunity by cotransduction of mouse dendritic cells with two genes encoding HER-2/neu and alpha tumor necrosis factor. *Cancer Gene Therapy*.

[32] Nimal S., Heath A., Thomas M. (2006). Enhancement of immune responses to an HIV gp120 DNA vaccine by fusion to TNF alpha cDNA. *Vaccine*.

[33] Belnoue E., Mayol J.-F., Carboni S. (2019). Targeting self- and neoepitopes with a modular self-adjuvanting cancer vaccine. *JCI Insight*.

[34] Belnoue E., Di Berardino-Besson W., Gaertner H. (2016). Enhancing antitumor immune responses by optimized combinations of cell-penetrating peptide-based vaccines and adjuvants. *Molecular Therapy*.

[35] Derouazi M., Di Berardino-Besson W., Belnoue E. (2015). Novel cell-penetrating peptide-based vaccine induces robust CD4+ and CD8+ T cell-mediated antitumor immunity. *Cancer Research*.

[36] Pouniotis D. S., Esparon S., Apostolopoulos V., Pietersz G. A. (2011). Whole protein and defined CD8^+^ and CD4^+^ peptides linked to penetratin targets both MHC class I and II antigen presentation pathways. *Immunology & Cell Biology*.

[37] Wu H., Zhuang Q., Xu J. (2019). Cell-penetrating peptide enhanced antigen presentation for cancer immunotherapy. *Bioconjugate Chemistry*.

[38] Brooks N., Esparon S., Pouniotis D., Pietersz G. (2015). Comparative immunogenicity of a cytotoxic T cell epitope delivered by penetratin and TAT cell penetrating peptides. *Molecules*.

[39] Brooks N., Hsu J., Esparon S., Pouniotis D., Pietersz G. (2018). Immunogenicity of a tripartite cell penetrating peptide containing a MUC1 variable number of tandem repeat (VNTR) and AT helper epitope. *Molecules*.

[40] Batchu R. B., Gruzdyn O., Potti R. B., Weaver D. W., Gruber S. A. (2014). MAGE-A3 with cell-penetrating domain as an efficient therapeutic cancer vaccine. *JAMA Surgery*.

[41] Granadillo M., Vallespi M. G., Batte A. (2011). A novel fusion protein-based vaccine comprising a cell penetrating and immunostimulatory peptide linked to human papillomavirus (HPV) type 16 E7 antigen generates potent immunologic and anti-tumor responses in mice. *Vaccine*.

[42] Pouniotis D., Tang C.-K., Apostolopoulos V., Pietersz G. (2016). Vaccine delivery by penetratin: mechanism of antigen presentation by dendritic cells. *Immunologic Research*.

[43] Pouniotis D. S., Apostolopoulos V., Pietersz G. A. (2006). Penetratin tandemly linked to a CTL peptide induces anti-tumour T-cell responses via a cross-presentation pathway. *Immunology*.

[44] Jorgovanovic D., Song M., Wang L., Zhang Y. (2020). Roles of IFN-*γ* in tumor progression and regression: a review. *Biomarker Research*.

[45] Dhanda S. K., Vir P., Raghava G. P. S. (2013). Designing of interferon-gamma inducing MHC class-II binders. *Biology Direct*.

[46] Dey J., Mahapatra S. R., Lata S., Patro S., Misra N., Suar M. (2022). Exploring *Klebsiella pneumoniae* capsule polysaccharide proteins to design multiepitope subunit vaccine to fight against pneumonia. *Expert Review of Vaccines*.

[47] Gupta S., Kapoor P., Chaudhary K., Gautam A., Kumar R., Patterson R. L. (2013). In silico approach for predicting toxicity of peptides and proteins. *PLOS ONE*.

[48] Colovos C., Yeates T. O. (1993). Verification of protein structures: patterns of nonbonded atomic interactions. *Protein Science*.

[49] Eisenberg D., Lüthy R., Bowie J. (1997). VERIFY3D: assessment of protein models with three-dimensional profiles. *Methods in Enzymology*.

[50] Lengauer T., Rarey M. (1996). Computational methods for biomolecular docking. *Current Opinion in Structural Biology*.

[51] Kozakov D., Hall D. R., Xia B. (2017). The ClusPro web server for protein–protein docking. *Nature Protocols*.

[52] Wallace A. C., Laskowski R. A., Thornton J. M. (1995). LIGPLOT: a program to generate schematic diagrams of protein–ligand interactions. *Protein Engineering, Design and Selection*.

[53] Pandey R. K., Verma P., Sharma D., Bhatt T. K., Sundar S., Prajapati V. K. (2016). High-throughput virtual screening and quantum mechanics approach to develop imipramine analogues as leads against trypanothione reductase of leishmania. *Biomedicine & Pharmacotherapy*.

[54] Tama F., Brooks C. L. (2006). Symmetry, form, and shape: guiding principles for robustness in macromolecular machines. *Annual Review of Biophysics and Biomolecular Structure*.

[55] López-Blanco J. R., Aliaga J. I., Quintana-Ortí E. S., Chacón P. (2014). iMODS: internal coordinates normal mode analysis server. *Nucleic Acids Research*.

[56] Lopéz-Blanco J. R., Garzón J. I., Chacón P. (2011). iMod: multipurpose normal mode analysis in internal coordinates. *Bioinformatics*.

[57] Abraham M. J., Murtola T., Schulz R. (2015). GROMACS: high performance molecular simulations through multi-level parallelism from laptops to supercomputers. *SoftwareX*.

[58] Grote A., Hiller K., Scheer M. (2005). JCat: a novel tool to adapt codon usage of a target gene to its potential expression host. *Nucleic Acids Research*.

[59] Bibi S., Ullah I., Zhu B. (2021). In silico analysis of epitope-based vaccine candidate against tuberculosis using reverse vaccinology. *Scientific Reports*.

[60] Rapin N., Lund O., Bernaschi M., Castiglione F., Brusic V. (2010). Computational immunology meets bioinformatics: the use of prediction tools for molecular binding in the simulation of the immune system. *PLOS ONE*.

[61] Schoenborn J. R., Wilson C. B. (2007). Regulation of interferon-*γ* during innate and adaptive immune responses. *Advances in Immunology*.

[62] Khan S., Khan A., Rehman A. U. (2019). Immunoinformatics and structural vaccinology driven prediction of multi-epitope vaccine against Mayaro virus and validation through in-silico expression. *Infection, Genetics and Evolution*.

[63] Zaib S., Akram F., Liaqat S. T. (2022). Bioinformatics approach for the construction of multiple epitope vaccine against omicron variant of SARS-CoV-2. *Scientific Reports*.

[64] Narang P. K., Dey J., Mahapatra S. R. (2021). Functional annotation and sequence-structure characterization of a hypothetical protein putatively involved in carotenoid biosynthesis in microalgae. *South African Journal of Botany*.

[65] Sudeshna Panda S., Dey J., Mahapatra S. R. (2022). Investigation on structural prediction of pectate lyase enzymes from different microbes and comparative docking studies with pectin: the economical waste from food industry. *Geomicrobiology Journal*.

[66] Mohammadzadeh Hosseini Moghri S. A. H., Chalbatani G. M., Ranjbar M., Raposo C., Abbasian A. (2023). CD171 multi-epitope peptide design based on immuno-informatics approach as a cancer vaccine candidate for glioblastoma. *Journal of Biomolecular Structure and Dynamics*.

[67] Behmard E., Abdulabbas H. T., Jasim S. A. (2022). Design of a novel multi-epitope vaccine candidate against hepatitis C virus using structural and nonstructural proteins: an immunoinformatics approach. *PLOS ONE*.

[68] Dey J., Mahapatra S. R., Raj T. K. (2022). Designing a novel multi-epitope vaccine to evoke a robust immune response against pathogenic multidrug-resistant enterococcus faecium bacterium. *Gut Pathogens*.

[69] Mahapatra S. R., Dey J., Jaiswal A., Roy R., Misra N., Suar M. (2022). Immunoinformatics-guided designing of epitope-based subunit vaccine from Pilus assembly protein of acinetobacter baumannii bacteria. *Journal of Immunological Methods*.

[70] T D., Kamaraj B., Vasudevan K. (2023). Structural immunoinformatics approach for rational design of a multi-epitope vaccine against triple negative breast cancer. *International Journal of Biological Macromolecules*.

[71] Schlom J. (2012). Therapeutic cancer vaccines: current status and moving forward. *JNCI Journal of the National Cancer Institute*.

[72] Sonmez M. G., Sönmez L. Ö. (2019). New treatment modalities with vaccine therapy in renal cell carcinoma. *Urology Annals*.

[73] Panowski S. H., Srinivasan S., Tan N. (2022). Preclinical development and evaluation of allogeneic CAR T cells targeting CD70 for the treatment of renal cell carcinoma. *Cancer Research*.

[74] Ramalingam P. S., Arumugam S. (2023). Reverse vaccinology and immunoinformatics approaches to design multi-epitope based vaccine against oncogenic KRAS. *Medical Oncology*.

[75] Tenzer S., Peters B., Bulik S. (2005). Modeling the MHC class I pathway by combining predictions of proteasomal cleavage, TAP transport and MHC class I binding. *Cellular and Molecular Life Sciences CMLS*.

[76] Fleri W., Paul S., Dhanda S. K. (2017). The immune epitope database and analysis resource in epitope discovery and synthetic vaccine design. *Frontiers in Immunology*.

[77] Damas M. S. F., Mazur F. G., Freire C. C. d. M., da Cunha A. F, da Silva Pranchevicius M.-C. (2022). A systematic immuno-informatic approach to design a multiepitope-based vaccine against emerging multiple drug resistant serratia marcescens. *Frontiers in Immunology*.

[78] Seyed N., Taheri T., Rafati S. (2016). Post-genomics and vaccine improvement for Leishmania. *Frontiers in Microbiology*.

[79] Samad A., Meghla N. S., Nain Z., Karpiński T. M., Rahman M. S. (2022). Immune epitopes identification and designing of a multi-epitope vaccine against bovine leukemia virus: a molecular dynamics and immune simulation approaches. *Cancer Immunology, Immunotherapy*.

[80] Michel-Todó L., Reche P. A., Bigey P., Pinazo M.-J., Gascón J., Alonso-Padilla J. (2019). In silico design of an epitope-based vaccine ensemble for chagas disease. *Frontiers in Immunology*.

[81] Meza B., Ascencio F., Sierra-Beltrán A. P., Torres J., Angulo C. (2017). A novel design of a multi-antigenic, multistage and multi-epitope vaccine against Helicobacter pylori: an in silico approach. *Infection, Genetics and Evolution*.

[82] Ruseska I., Zimmer A. (2020). Internalization mechanisms of cell-penetrating peptides. *Beilstein Journal of Nanotechnology*.

[83] Fathollahi M., Fathollahi A., Motamedi H., Moradi J., Alvandi A., Abiri R. (2021). In silico vaccine design and epitope mapping of New Delhi metallo-beta-lactamase (NDM): an immunoinformatics approach. *BMC Bioinformatics*.

[84] Obaidullah A. J., Alanazi M. M., Alsaif N. A. (2021). Immunoinformatics-guided design of a multi-epitope vaccine based on the structural proteins of severe acute respiratory syndrome coronavirus 2. *RSC Advances*.

[85] Mahapatra S. R., Dey J., Raj T. K. (2022). The potential of plant-derived secondary metabolites as novel drug candidates against Klebsiella pneumoniae: molecular docking and simulation investigation. *South African Journal of Botany*.

