# Supplementary material for: Immunoinformatics Approach to Design a Chimeric CD70-Peptide Vaccine against Renal Cell Carcinoma
Source: J Immunol Res. 2024 Jan 27;2024:2875635. doi: 10.1155/2024/2875635 (PMC10838208; doi:10.1155/2024/2875635)
Supplement: Supplementary Materials — Figure S1: that shows 2D structure of vaccine constructs, Figures S2–S5: that correspond to the refined and validated structure of vaccines and tumor necrosis factor receptor (TNFR), and Figure S6: representing Ligplot analysis showing the hydrogen bonding and hydrophobic interactions between TNFR and vaccine constructs. This file also includes Tables S1 and S2: which, respectively, contain predicted linear and discontinues epitopes of CD70 peptide vaccine constructs. [file 2875635.f1.docx]

**a)**

**
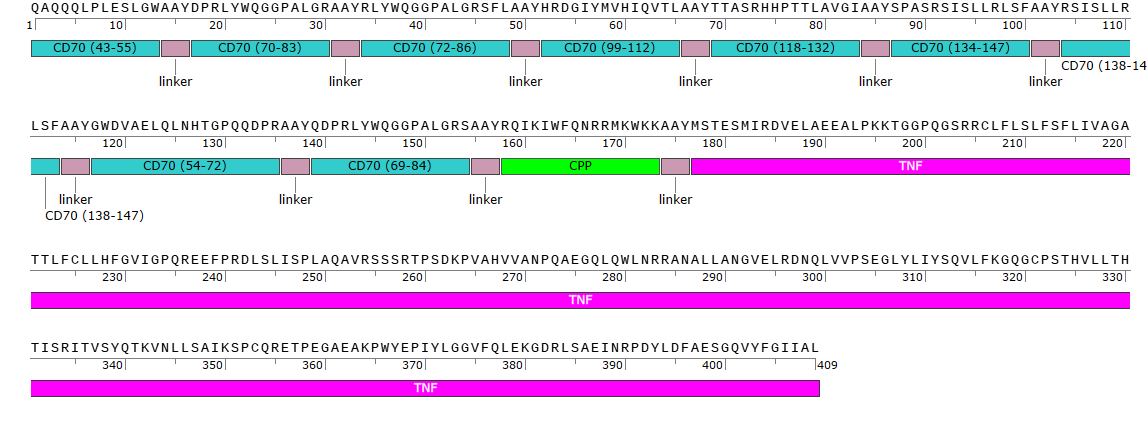
**

**b)**

**
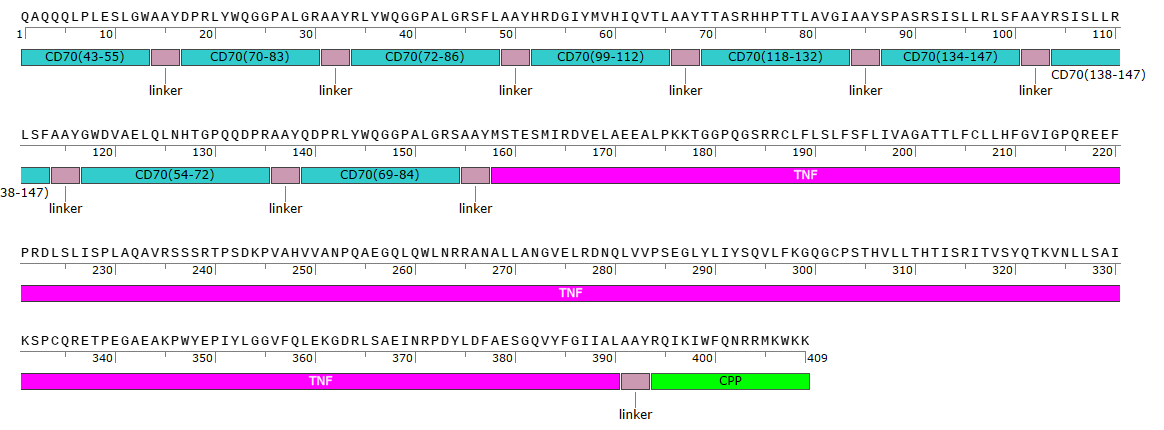
**

**c)**

**
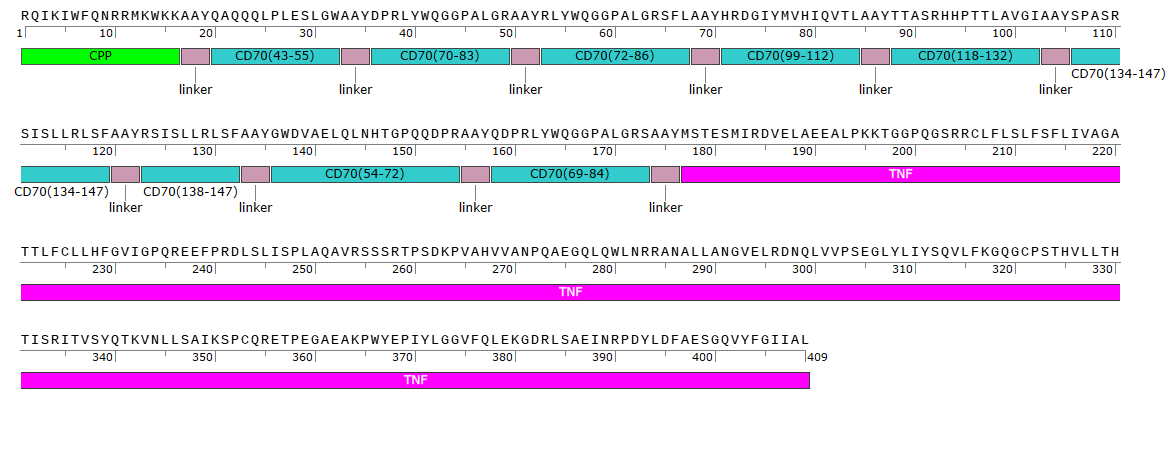
**

**d)**


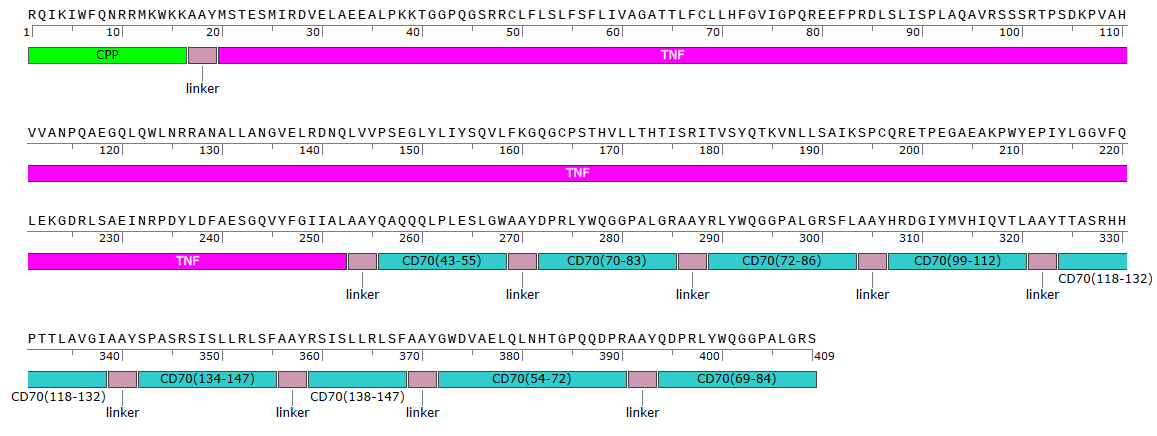


**e)**

**
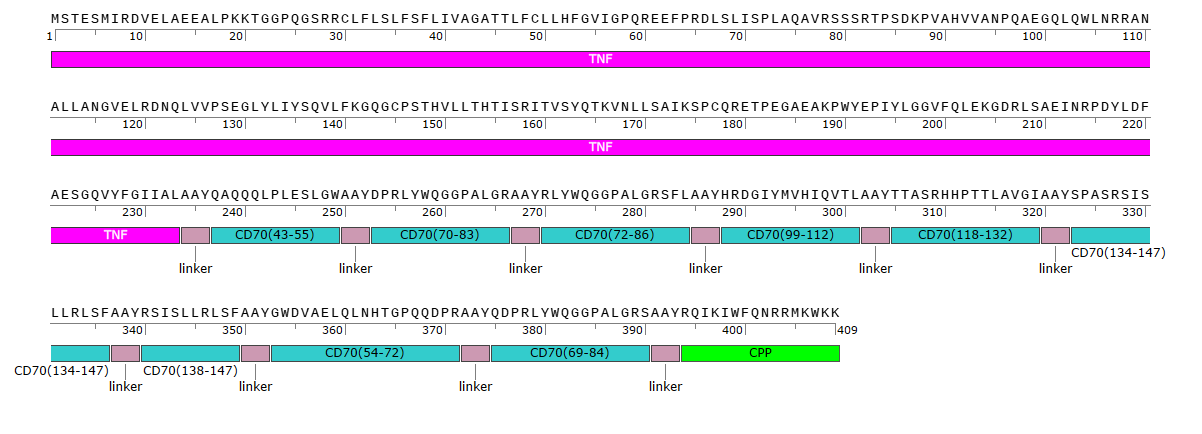
**

**f)**

**
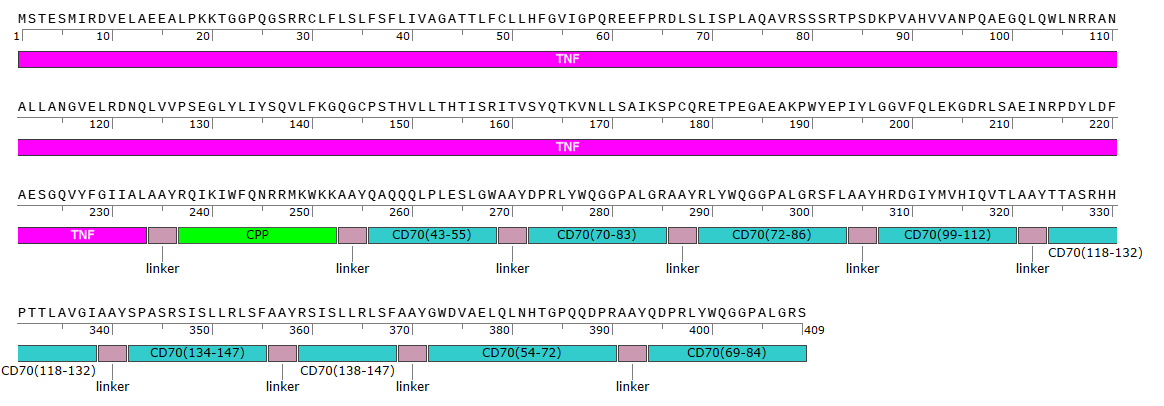
**

**Fig.S1. Homo sapiens CD70 vaccine constructs**. **a.** CD70**^(epitopes)^**-CPP- TNF **^(whole sequences)^**. **b.** CD70**^(epitopes)^**- TNF **^(whole sequences)^-**CPP. **c.** CPP- CD70**^(epitopes)^**- TNF **^(whole sequences)^**. **d.** CPP- TNF **^(whole sequences^** - CD70**^(epitopes)^**. **e.** TNF **^(whole sequences)^** - CD70**^(epitopes)^** –CPP. **f.** TNF **^(whole sequences)^** -CPP- CD70**^(epitopes)^**. All structures were designed utilizing the SnappGene®3.2.1 tool, incorporating the AAY sequence (alanine/alanine/tyrosine) as a proteolytic linker."

**a ) a)**

**
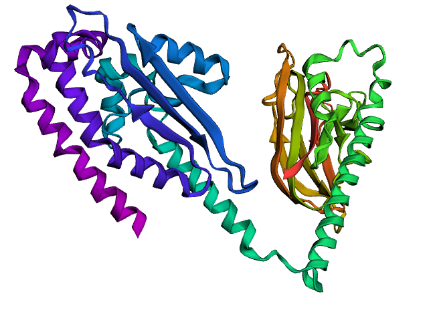

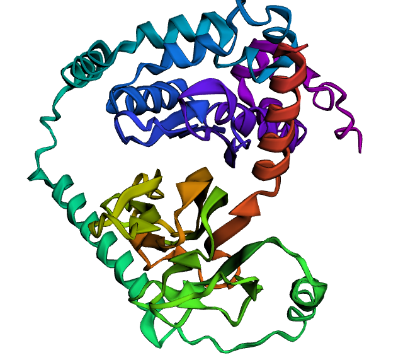
**

**
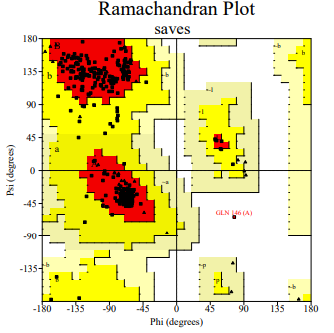

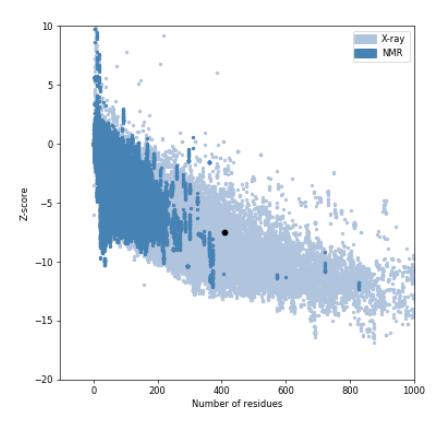
b) c ) b) c)**


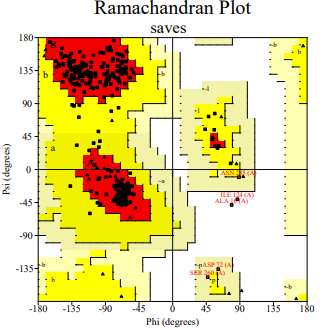

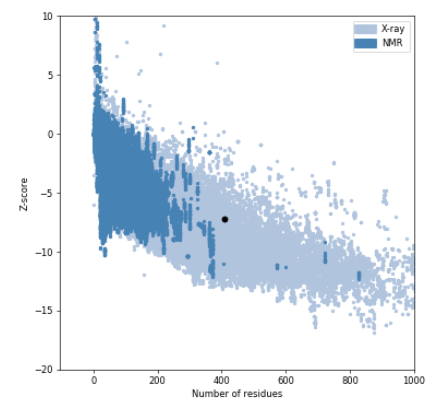


**
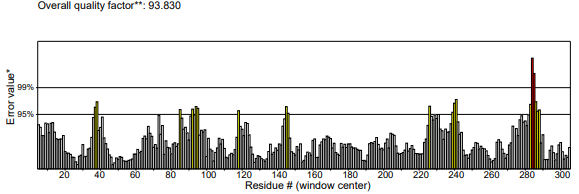

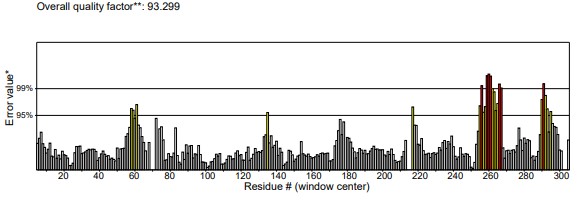
d) d)**

**e) e)**

**
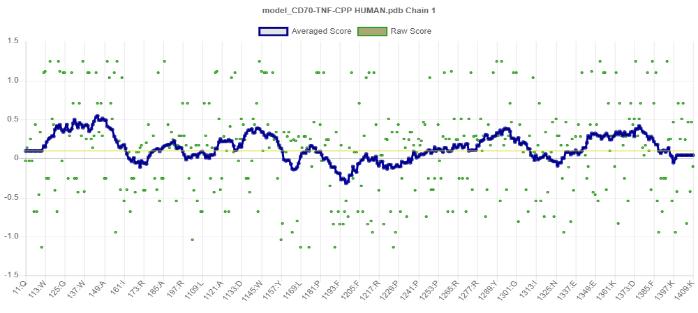

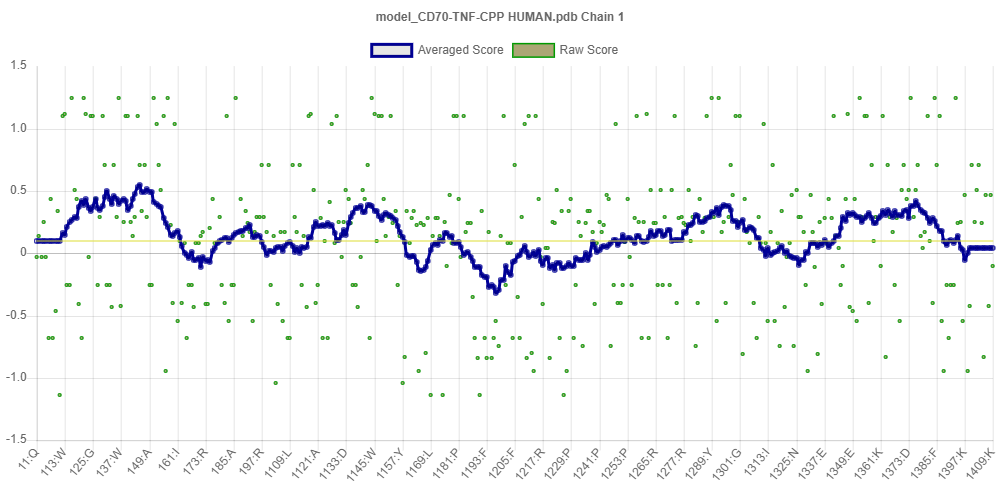
**

**Fig. S2.** The outcomes from diverse structure validation tools affirmed the reliability and precision of the CD70 **^(epitopes)^** -TNF ^(Whole sequence)^ -CPP [Homo sapiens] and CPP- CD70 **^(epitopes)^** -TNF ^(Whole sequence)^ [Homo sapiens] vaccine constructs. **Left picture** **a.** The 3D model of the CD70 **^(epitopes)^** -TNF ^(Whole sequence)^ -CPP [Homo sapiens] vaccine construct was developed. **b.** The ProSA-web z-score plot displayed a Z-score = − 7.54. **c.** The Ramachadran plot exhibited 92.9% of residues in the most favored regions. **d.** The ERRAT overall quality factor exceeded 93%. **e.** Post-refinement, the Verify 3D indicated a score of 58.44% for the model. **Right picture** **a.** The 3D model of the CPP- CD70 **^(epitopes)^** -TNF ^(Whole sequence)^ [Homo sapiens] vaccine construct was developed. **b.** The ProSA-web z-score plot displayed a Z-score = − 7.2. **c.** The Ramachadran plot exhibited 92.6% of residues in the most favored regions. **d.** The ERRAT overall quality factor exceeded 93%. **e.** Post-refinement, the Verify 3D indicated a score of 70.8% for the model.


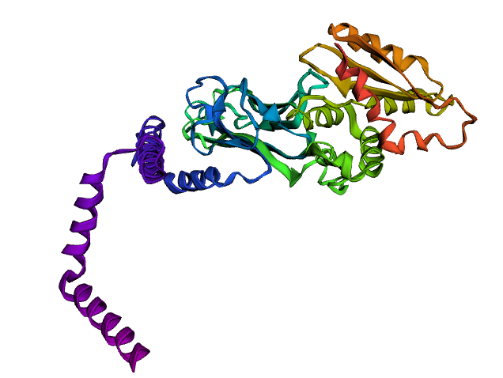
**a ) a)**


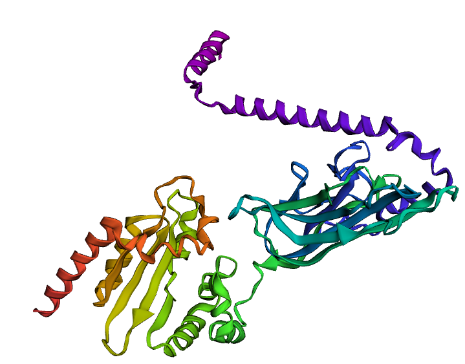


**b) c) b) c)**


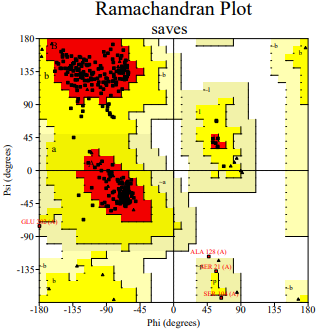

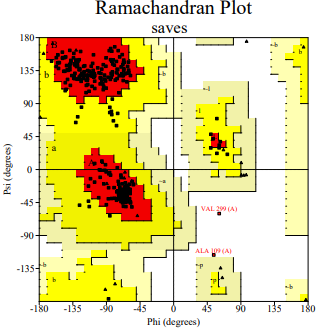

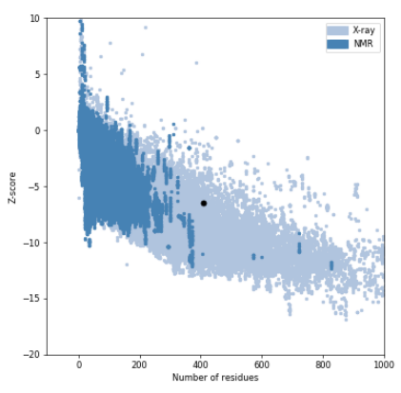

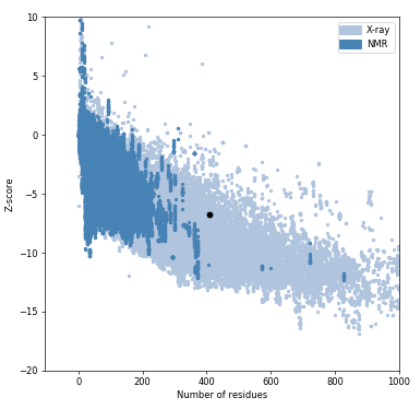


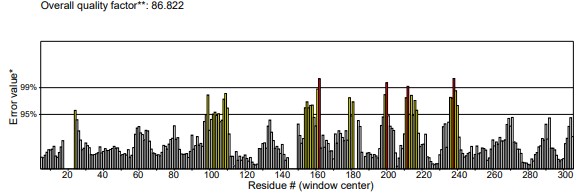

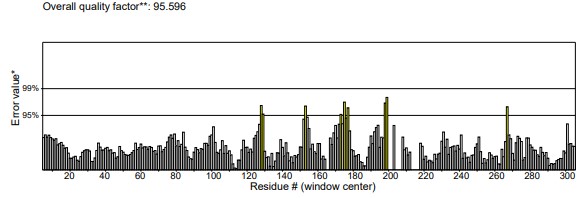
**d)** **d)**

**e) e)**

**
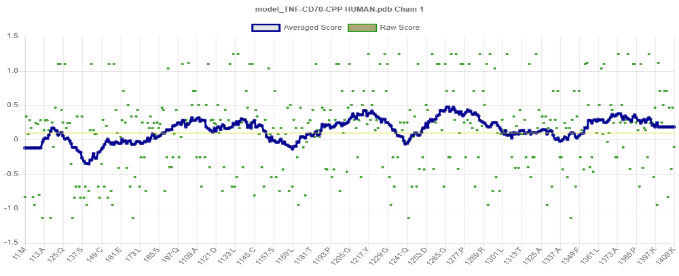

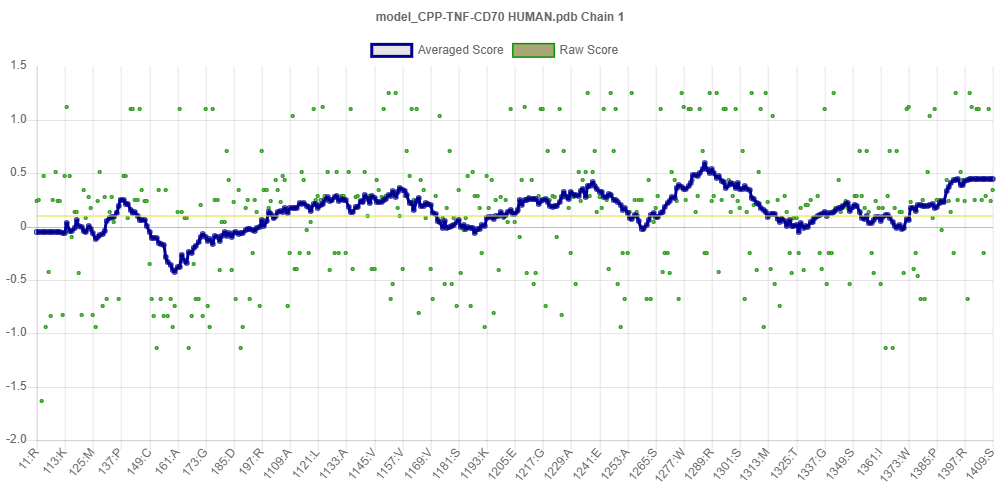
**

**Fig. S3**. The outcomes from diverse structure validation tools affirmed the reliability and precision of the CPP-TNF ^(Whole sequence)^ - CD70 **^(epitopes)^** [Homo sapiens] and TNF ^(Whole sequence)^ - CD70 **^(epitopes)^** -CPP [Homo sapiens] vaccine constructs. **Left picture** **a.** The 3D model of the CPP-TNF ^(Whole sequence)^ - CD70 **^(epitopes)^** [Homo sapiens] vaccine construct was developed. **b.** The ProSA-web z-score plot displayed a Z-score = − 6.75. **c.** The Ramachadran plot exhibited 93.2% of residues in the most favored regions. **d.** The ERRAT overall quality factor exceeded 95%. **e.** Post-refinement, the Verify 3D indicated a score of 60.88% for the model. **Right picture** **a.** The 3D model of the TNF ^(Whole sequence)^ - CD70 **^(epitopes)^** -CPP [Homo sapiens] vaccine construct was developed. **b.** The ProSA-web z-score plot displayed a Z-score = − 6.48. **c.** The Ramachadran plot exhibited 93.2% of residues in the most favored regions. **d.** The ERRAT overall quality factor exceeded 86%. **e.** Post-refinement, the Verify 3D indicated a score of 63.08% for the model.

**
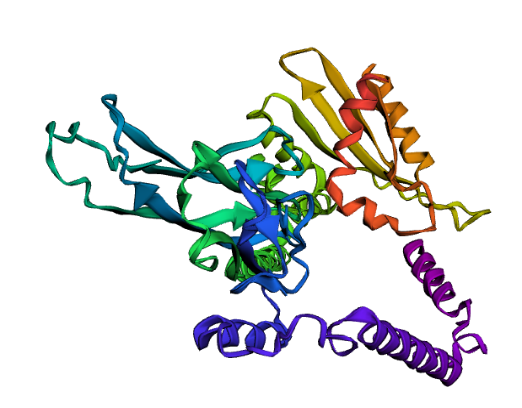
**

**a)**


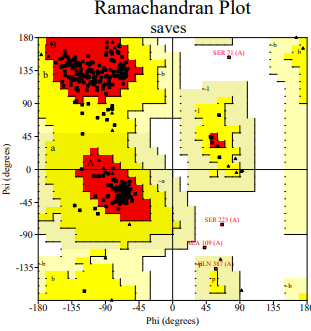

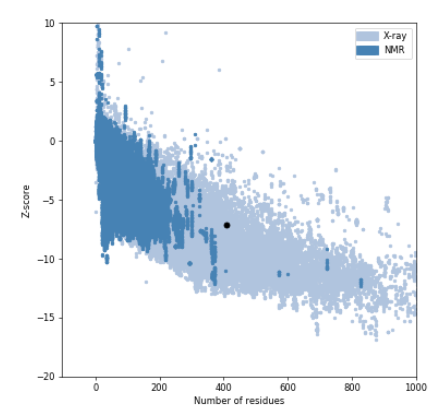
 **b) c)**

**d) e)**


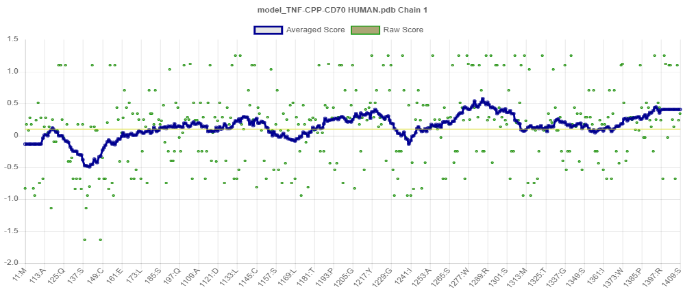

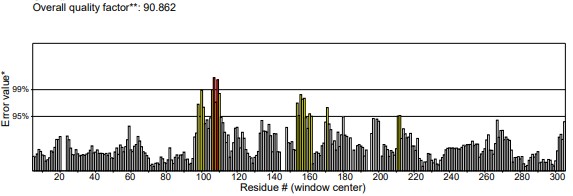


**Fig. S4.** The outcomes from diverse structure validation tools affirmed the reliability and precision of the TNF ^(Whole sequence)^ –CPP- CD70 **^(epitopes)^** [Homo sapiens] vaccine construct **a.** The 3D model of the TNF ^(Whole sequence)^ –CPP- CD70 **^(epitopes)^** [Homo sapiens] vaccine construct. **b.** The ProSA-web z-score plot displayed a Z-score = − 7.1. **c.** The Ramachadran plot exhibited 93.2% of residues in the most favored regions. **d.** The ERRAT overall quality factor exceeded 90%. **e.** Post-refinement, the Verify 3D indicated a score of 64.30% for the model.


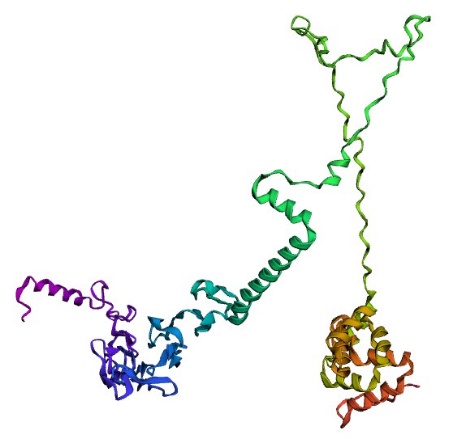
 **a)**


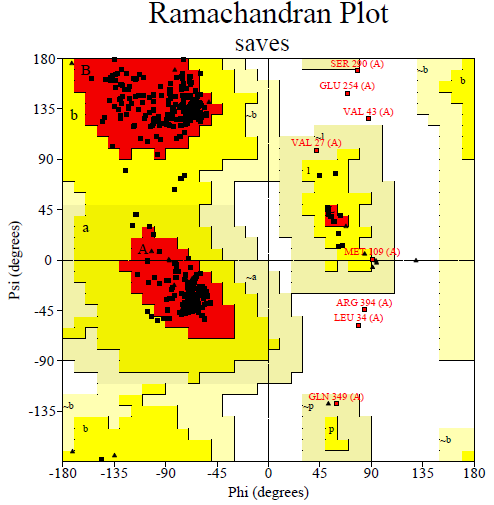

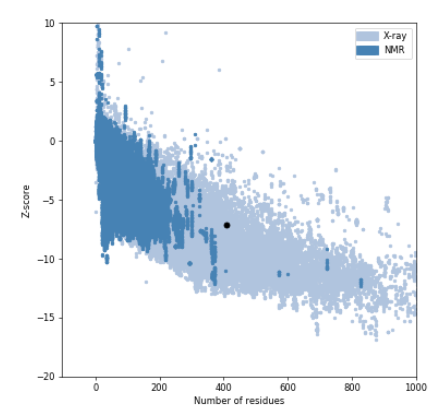
 **b) c)**


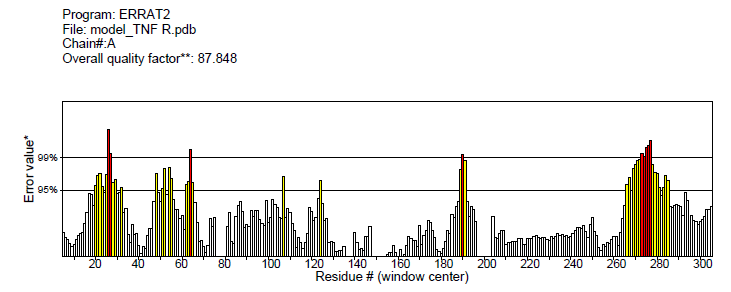
 **d) e)**

**
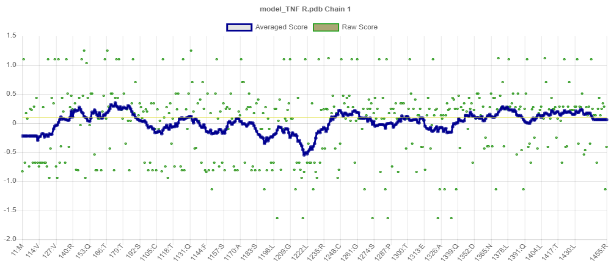
**

**Fig. S5.** The outcomes from diverse structure validation tools affirmed the reliability and precision of the TNF receptor **a.** The 3D model of the TNF receptor. **b.** The ProSA-web z-score plot displayed a Z-score = − 6.72. **c.** The Ramachadran plot exhibited 93.8% of residues in the most favored regions. **d.** The ERRAT overall quality factor exceeded 87%. **e.** Post-refinement, the Verify 3D indicated a score of 42.07% for the model.

| **a)**  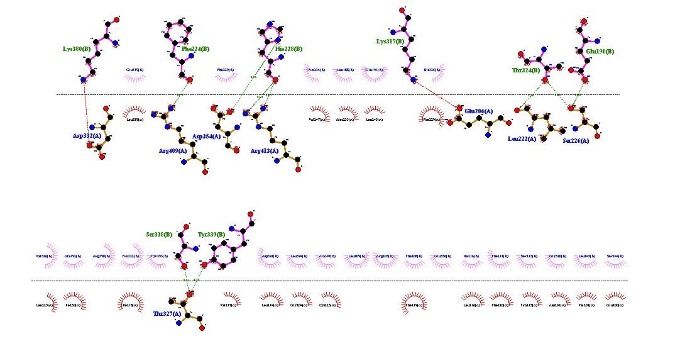 | **b)**  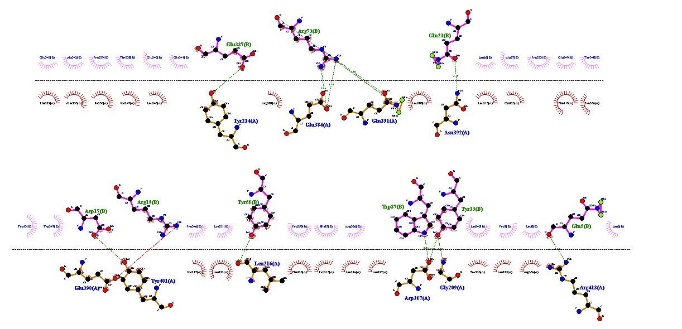 |
| --- | --- |
| **c)** 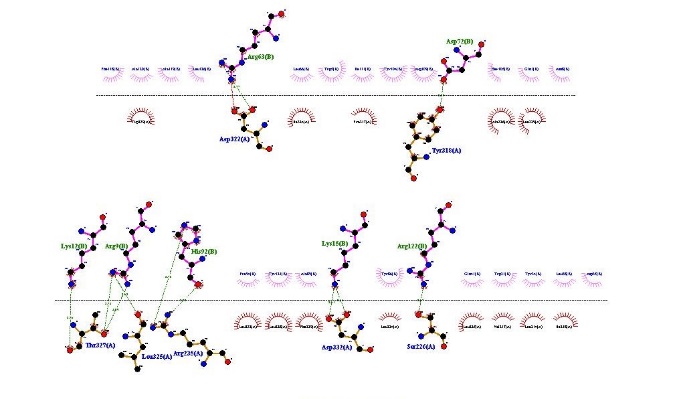 | **d)** 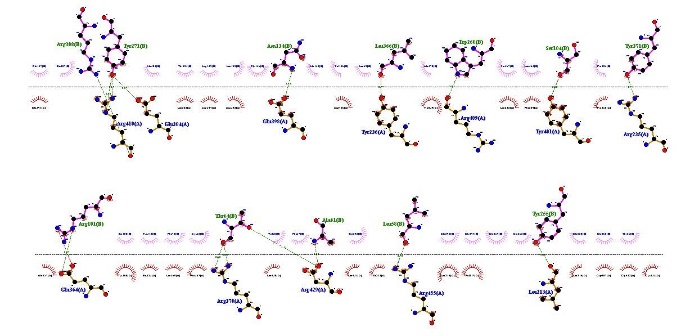 |
| **e)** 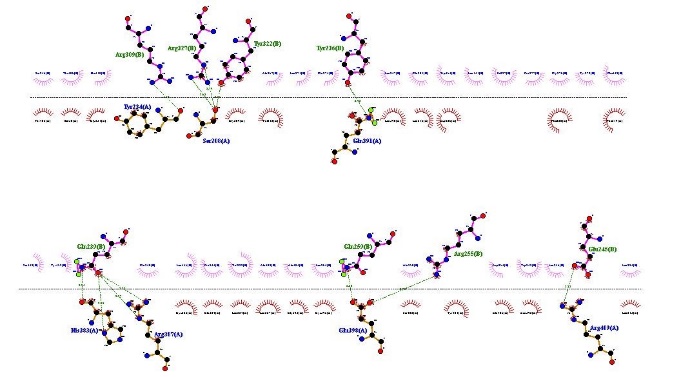 | **f** ) 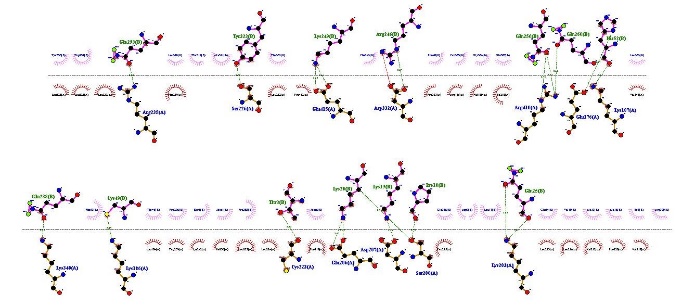 |
| **g)** 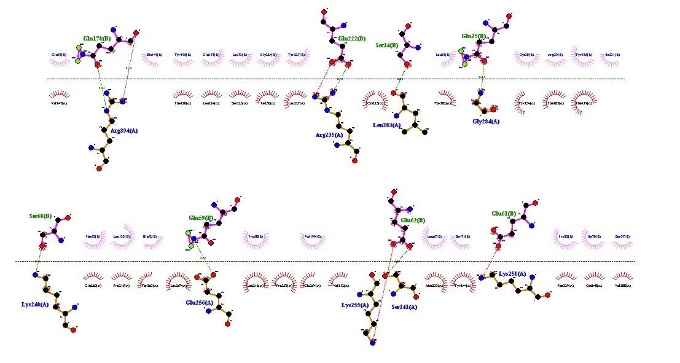 | 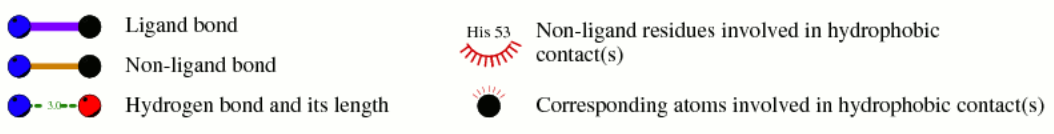 |

**Fig. S6.** Ligplot analysis showing the hydrogen bonding and hydrophobic interactions between tumor necrosis factor receptor (TNFR) and vaccine constructs. The figures depict binding between TNFR and **a.** CD70-CPP-TNF **b.** CD70-TNF-CPP **c.** CPP-CD70-TNF **d.** CPP-TNF-CD70 **e.** TNF-CD70-CPP **f.** TNF-CPP-CD70 **g.** TNF. Dashed red lines represent salt bridge interactions, while dashed green lines indicate hydrogen bonds. Because this is a two-dimensional representation, actual distances, bond lengths, and spatial organization are distorted.

**Table S1.** Predicted linear epitopes of CD70 [Homo sapiens] peptide vaccine constructs.

| **Construct** | **No.** | **Chain** | **Start** | **End** | **Peptide** | **Number of residues** | **Score** |
| --- | --- | --- | --- | --- | --- | --- | --- |
| CD70-TNF-CPP  [Homo sapiens] | 1 | A | 148 | 244 | GPALGRSAAYMSTESMIRDVELAEEALPKKTGGPQGSRRCLFLSLFSFLIVAGATTLFCLLHFGVIGPQREEFPRDLSLISPLAQAVRSSSRTPSDK | 97 | 0.789 |
|  | 2 | A | 1 | 20 | QAQQQLPLESLGWAAYDPRL | 20 | 0.769 |
|  | 3 | A | 127 | 140 | HTGPQQDPRAAYQD | 14 | 0.678 |
|  | 4 | A | 316 | 323 | ITVSYQTK | 8 | 0.66 |
|  | 5 | A | 336 | 346 | RETPEGAEAKP | 11 | 0.643 |
|  | 6 | A | 88 | 91 | PASR | 4 | 0.619 |
|  | 7 | A | 360 | 364 | EKGDR | 5 | 0.613 |
|  | 8 | A | 269 | 279 | LLANGVELRDN | 11 | 0.573 |
|  | 9 | A | 282 | 286 | VVPSE | 5 | 0.565 |
|  | 10 | A | 262 | 266 | LNRRA | 5 | 0.562 |
|  | 11 | A | 69 | 75 | TTASRHH | 7 | 0.549 |
|  | 12 | A | 253 | 259 | PQAEGQL | 7 | 0.511 |
| CPP-CD70-TNF  [Homo sapiens] | 1 | A | 346 | 368 | SAIKSPCQRETPEGAEAKPWYEP | 23 | 0.868 |
|  | 2 | A | 315 | 328 | FKGQGCPSTHVLLT | 14 | 0.833 |
|  | 3 | A | 188 | 214 | AEEALPKKTGGPQGSRRCLFLSLFSFL | 27 | 0.819 |
|  | 4 | A | 82 | 96 | TLAAYTTASRHHPTT | 15 | 0.76 |
|  | 5 | A | 1 | 52 | QIKIWFQNRRMKWKKAAYQAQQQLPLESLGWAAYDPRLYWQGGPALGRAAYR | 52 | 0.72 |
|  | 6 | A | 334 | 343 | ITVSYQTKVN | 10 | 0.718 |
|  | 7 | A | 231 | 246 | IGPQREEFPRDLSLIS | 16 | 0.695 |
|  | 8 | A | 117 | 126 | SFAAYRSISL | 10 | 0.666 |
|  | 9 | A | 106 | 111 | PASRSI | 6 | 0.587 |
|  | 10 | A | 388 | 397 | NRPDYLDFAE | 10 | 0.581 |
|  | 11 | A | 270 | 276 | NPQAEGQ | 7 | 0.549 |
|  | 12 | A | 376 | 381 | QLEKGD | 6 | 0.533 |
| CPP-TNF-CD70  [Homo sapiens] | 1 | A | 1 | 56 | RQIKIWFQNRRMKWKKAAYMSTESMIRDVELAEEALPKKTGGPQGSRRCLFLSLFS | 56 | 0.885 |
|  | 2 | A | 191 | 212 | AIKSPCQRETPEGAEAKPWYEP | 22 | 0.806 |
|  | 3 | A | 158 | 172 | LFKGQGCPSTHVLLT | 15 | 0.75 |
|  | 4 | A | 322 | 336 | AYTTASRHHPTTLAV | 15 | 0.704 |
|  | 5 | A | 343 | 398 | PASRSISLLRLSFAAYRSISLLRLSFAAYGWDVAELQLNHTGPQQDPRAAYQDPRL | 56 | 0.675 |
|  | 6 | A | 401 | 409 | QGGPALGRS | 9 | 0.635 |
|  | 7 | A | 232 | 241 | NRPDYLDFAE | 10 | 0.614 |
|  | 8 | A | 76 | 91 | GPQREEFPRDLSLISP | 16 | 0.586 |
|  | 9 | A | 114 | 121 | NPQAEGQL | 8 | 0.528 |
| TNF-CD70-CPP  [Homo sapiens] | 1 | A | 1 | 41 | MSTESMIRDVELAEEALPKKTGGPQGSRRCLFLSLFSFLIV | 41 | 0.817 |
|  | 2 | A | 173 | 193 | IKSPCQRETPEGAEAKPWYEP | 21 | 0.813 |
|  | 3 | A | 141 | 152 | KGQGCPSTHVLL | 12 | 0.804 |
|  | 4 | A | 384 | 409 | GPALGRSAAYRQIKIWFQNRRMKWKK | 26 | 0.782 |
|  | 5 | A | 320 | 330 | AAYSPASRSIS | 11 | 0.768 |
|  | 6 | A | 44 | 85 | ATTLFCLLHFGVIGPQREEFPRDLSLISPLAQAVRSSSRTPS | 42 | 0.678 |
|  | 7 | A | 276 | 293 | GPALGRSFLAAYHRDGIY | 18 | 0.648 |
|  | 8 | A | 343 | 359 | SLLRLSFAAYGWDVAEL | 17 | 0.639 |
|  | 9 | A | 239 | 249 | QQQLPLESLGW | 11 | 0.577 |
|  | 10 | A | 213 | 222 | NRPDYLDFAE | 10 | 0.525 |
| TNF-CPP-CD70  [Homo sapiens] | 1 | A | 173 | 193 | IKSPCQRETPEGAEAKPWYEP | 21 | 0.84 |
|  | 2 | A | 1 | 72 | MSTESMIRDVELAEEALPKKTGGPQGSRRCLFLSLFSFLIVAGATTLFCLLHFGVIGPQREEFPRDLSLISP | 72 | 0.805 |
|  | 3 | A | 140 | 152 | FKGQGCPSTHVLL | 13 | 0.805 |
|  | 4 | A | 339 | 350 | AAYSPASRSISL | 12 | 0.761 |
|  | 5 | A | 362 | 375 | SLLRLSFAAYGWDV | 14 | 0.746 |
|  | 6 | A | 319 | 332 | TLAAYTTASRHHPT | 14 | 0.667 |
|  | 7 | A | 260 | 268 | QLPLESLGW | 9 | 0.638 |
|  | 8 | A | 293 | 314 | QGGPALGRSFLAAYHRDGIYMV | 22 | 0.63 |
|  | 9 | A | 213 | 218 | NRPDYL | 6 | 0.598 |
|  | 10 | A | 96 | 102 | PQAEGQL | 7 | 0.527 |

**Table S2.** Predicted discontinuous epitopes of CD70 ligand [Homo sapiens] peptide vaccine constructs

| **Construct** | **No.** | **Residues** | **Number of residues** | **Score** |
| --- | --- | --- | --- | --- |
| CD70-TNF-CPP  [Homo sapiens] | 1 | A:M158, A:S159, A:T160, A:E161, A:S162, A:M163, A:I164, A:R165, A:D166, A:V167, A:E168, A:L169, A:A170, A:E171, A:E172, A:A173, A:L174, A:P175, A:K176 | 19 | 0.804 |
|  | 2 | A:H52, A:A391, A:R394, A:Q395, A:I396, A:K397, A:I398, A:W399, A:F400, A:Q401, A:N402, A:R403, A:R404, A:M405, A:W407, A:K408, A:K409 | 17 | 0.774 |
|  | 3 | A:K177, A:T178, A:G179, A:G180, A:P181, A:Q182, A:G183, A:S184, A:R185, A:R186, A:C187, A:L188, A:F189, A:L190, A:S191, A:L192, A:F193, A:S194, A:F195, A:L196, A:I197, A:V198, A:A199, A:G200, A:A201, A:T202, A:T203, A:L204, A:C206, A:L207, A:L208, A:H209, A:F210, A:G211, A:V212, A:I213, A:G214, A:P215, A:Q216, A:R217, A:N252, A:P253, A:Q254, A:A255, A:E256, A:G257, A:Q258, A:L259, A:Q260, A:Q300, A:G301, A:C302, A:P303, A:S304, A:T305, A:R336, A:E337, A:T338, A:P339, A:E340, A:G341, A:A342, A:E343, A:A344, A:K345, A:P346 | 66 | 0.726 |
|  | 4 | A:Q1, A:A2, A:Q3, A:Q4, A:Q5, A:L6, A:P7, A:L8, A:E9, A:S10, A:L11, A:G12, A:W13, A:A14, A:A15, A:Y16, A:D17, A:P18, A:R19, A:L20, A:G24, A:P26, A:A27, A:T69, A:T70, A:A71, A:S72, A:R73, A:H74, A:H75, A:L125, A:T128, A:G129, A:P130, A:Q131, A:Q132, A:D133, A:P134, A:R135, A:A136, A:A137, A:Y138, A:Q139, A:D140, A:Y144 | 45 | 0.689 |
|  | 5 | A:E218, A:F220, A:P221, A:R222, A:D223, A:L224, A:S225, A:L226, A:I227, A:S228, A:P229, A:L230, A:A231, A:Q232, A:A233, A:V234, A:R235, A:S236, A:S237, A:S238, A:R239, A:T240, A:P241, A:S242, A:D243, A:K244, A:P245, A:L262, A:N263, A:R264, A:N267, A:A268, A:L269, A:L270, A:A271, A:N272, A:G273, A:V274, A:E275, A:R277, A:V282, A:V283, A:P284, A:S285, A:E286, A:I316, A:T317, A:V318, A:S319, A:Y320, A:Q321, A:T322, A:K323, A:E360, A:K361, A:G362, A:D363, A:R364 | 58 | 0.673 |
|  | 6 | A:G148, A:P149, A:A150, A:G152, A:R153, A:S154, A:A156, A:Y157 | 8 | 0.65 |
|  | 7 | A:R53, A:D54, A:G55, A:P88, A:A89, A:S90, A:R91 | 7 | 0.583 |
| CPP-CD70-TNF  [Homo sapiens] | 1 | A:A188, A:E189, A:E190, A:A191, A:L192, A:P193, A:K194, A:K195, A:T196, A:G197, A:G198, A:P199, A:Q200, A:G201, A:S202, A:R203, A:R204, A:C205, A:L206, A:F207, A:L208, A:S209, A:L210, A:F211 | 24 | 0.843 |
|  | 2 | A:N270, A:P271, A:Q272, A:A273, A:E274, A:G275, A:Q276, A:Q278, A:F315, A:K316, A:G317, A:Q318, A:G319, A:C320, A:P321, A:S322, A:T323, A:H324, A:V325, A:L326, A:L327, A:T328, A:S346, A:A347, A:I348, A:K349, A:S350, A:P351, A:C352, A:Q353, A:R354, A:E355, A:T356, A:P357, A:E358, A:G359, A:A360, A:E361, A:A362, A:K363, A:P364, A:W365, A:Y366, A:E367, A:P368, A:N388, A:R389, A:P390, A:D391, A:Y392, A:L393, A:D394, A:F395, A:A396, A:E397, A:Q400 | 56 | 0.75 |
|  | 3 | A:A16, A:A17, A:Y18, A:Q19, A:A20, A:Q21, A:Q22, A:Q23, A:L24, A:P25, A:L26, A:E27, A:S28, A:L29, A:G30, A:W31, A:A32, A:A33, A:Y34, A:D35, A:P36, A:R37, A:L38, A:Y39, A:Q41, A:G42, A:G43, A:P44, A:A45, A:L46, A:G47, A:R48, A:A49, A:R52, A:W55, A:Q56, A:V81, A:T82, A:L83, A:A84, A:A85, A:Y86, A:T87, A:T88, A:A89, A:S90, A:R91, A:H93, A:P94, A:T95, A:T96, A:S117, A:F118, A:A119, A:A120, A:Y121, A:I124, A:S125, A:L126, A:L129, A:S130, A:A133, A:Y134 | 63 | 0.732 |
|  | 4 | A:Q1, A:I2, A:K3, A:I4, A:W5, A:Q7, A:N8, A:R9, A:M11, A:K12, A:R63 | 11 | 0.677 |
|  | 5 | A:G229, A:I231, A:G232, A:P233, A:R235, A:E236, A:E237, A:F238, A:D241, A:L242, A:S243, A:L244, A:I245, A:S246, A:R282 | 15 | 0.654 |
|  | 6 | A:V301, A:S303, A:E304, A:I334, A:T335, A:V336, A:S337, A:Y338, A:Q339, A:T340, A:K341, A:V342, A:N343, A:Q376, A:E378, A:K379, A:G380, A:D381 | 18 | 0.619 |
|  | 7 | A:C224, A:H227, A:F228 | 3 | 0.618 |
|  | 8 | A:S105, A:P106, A:A107, A:S108, A:R109, A:S110, A:I111 | 7 | 0.557 |
| CPP-TNF-CD70  [Homo sapiens] | 1 | A:R1, A:Q2, A:I3, A:K4, A:I5, A:W6, A:F7, A:Q8, A:N9, A:R10, A:R11, A:M12, A:W14, A:K15, A:A17, A:A18, A:Y19, A:M20, A:S21, A:T22, A:E23, A:S24, A:M25, A:I26, A:R27, A:D28, A:V29, A:E30, A:L31, A:A32, A:E33, A:E34, A:A35, A:L36, A:P37 | 35 | 0.948 |
|  | 2 | A:K38, A:K39, A:T40, A:G41, A:G42, A:P43, A:Q44, A:G45, A:S46, A:R47, A:R48, A:C49, A:L50, A:F51, A:L52, A:S53, A:L54, A:F55, A:S56, A:I59, A:V60, A:A63 | 22 | 0.73 |
|  | 3 | A:N114, A:P115, A:Q116, A:A117, A:E118, A:G119, A:Q120, A:L121, A:Q122, A:L158, A:F159, A:K160, A:G161, A:Q162, A:G163, A:C164, A:P165, A:S166, A:T167, A:H168, A:V169, A:L170, A:L171, A:T172, A:A191, A:I192, A:K193, A:S194, A:P195, A:C196, A:Q197, A:R198, A:E199, A:T200, A:P201, A:E202, A:G203, A:A204, A:E205, A:A206, A:K207, A:P208, A:W209, A:Y210, A:E211, A:P212, A:N232, A:R233, A:P234, A:D235, A:Y236, A:L237, A:D238, A:F239, A:A240, A:E241 | 56 | 0.709 |
|  | 4 | A:G280, A:P281, A:R285, A:I316, A:A322, A:Y323, A:T324, A:T325, A:A326, A:S327, A:R328, A:H329, A:H330, A:P331, A:T332, A:T333, A:L334, A:A335, A:V336, A:P343, A:A344, A:S345, A:R346, A:S347, A:I348, A:S349, A:L350, A:L351, A:R352, A:L353, A:S354, A:F355, A:A356, A:A357, A:Y358, A:R359, A:S360, A:I361, A:S362, A:L363, A:L364, A:R365, A:L366, A:S367, A:A369, A:A370, A:Y371, A:G372, A:W373, A:D374, A:V375, A:A376, A:E377, A:L378, A:Q379, A:L380, A:N381, A:H382, A:T383, A:G384, A:P385, A:Q386, A:Q387, A:D388, A:P389, A:R390, A:A391, A:A392, A:Y393, A:Q394, A:D395, A:P396, A:R397, A:L398, A:G402, A:G403, A:P404, A:A405, A:L406, A:G407, A:R408, A:S409 | 82 | 0.668 |
|  | 5 | A:F82, A:P83, A:D85, A:L86, A:S87, A:L88, A:I89, A:S90, A:P91 | 9 | 0.631 |
| TNF-CD70-CPP  [Homo sapiens] | 1 | A:M1, A:S2, A:T3, A:E4, A:S5, A:M6, A:I7, A:R8, A:D9, A:V10, A:E11, A:L12, A:A13, A:E14, A:E15, A:A16, A:L17, A:P18 | 18 | 0.932 |
|  | 2 | A:K141, A:G142, A:Q143, A:G144, A:C145, A:P146, A:S147, A:T148, A:H149, A:V150, A:L151, A:L152, A:I173, A:K174, A:S175, A:P176, A:C177, A:Q178, A:R179, A:E180, A:T181, A:P182, A:E183, A:G184, A:A185, A:E186, A:A187, A:K188, A:P189, A:W190, A:Y191, A:E192, A:P193, A:L218, A:D219, A:F220, A:A221, A:E222 | 38 | 0.769 |
|  | 3 | A:Q239, A:Q240, A:Q241, A:L242, A:P243, A:L244, A:E245, A:S246, A:G248, A:W249, A:Y252, A:D253, A:P254, A:R255, A:W258, A:G276, A:P277, A:A278, A:G280, A:R281, A:S282, A:L284, A:A285, A:A286, A:Y287, A:H288, A:R289, A:D290, A:G291, A:I292, A:Y293, A:A320, A:A321, A:Y322, A:S323, A:P324, A:A325, A:S326, A:R327, A:S328, A:I329, A:S330, A:Y339, A:S343, A:L344, A:R346, A:L347, A:S348, A:F349, A:A350, A:A351, A:Y352, A:G353, A:W354, A:D355, A:V356, A:A357, A:E358, A:L359, A:Q360, A:G384, A:P385, A:A386, A:L387, A:G388, A:R389, A:S390, A:A391, A:A392, A:Y393, A:R394, A:Q395, A:I396, A:K397, A:I398, A:W399, A:F400, A:Q401, A:N402, A:R403, A:R404, A:M405, A:K406, A:W407 | 84 | 0.692 |
|  | 4 | A:K19, A:K20, A:T21, A:G22, A:G23, A:P24, A:Q25, A:G26, A:S27, A:R28, A:R29, A:C30, A:L31, A:F32, A:L33, A:S34, A:L35, A:F36, A:S37, A:F38, A:I40, A:V41, A:A44, A:T45, A:L47, A:F48, A:C49, A:L50, A:L51, A:H52, A:F53, A:G54, A:V55, A:I56, A:G57, A:P58, A:Q59, A:E62, A:F63, A:D66, A:L67, A:S68, A:L69, A:I70, A:S71, A:P72, A:L73, A:A74, A:Q75, A:A76, A:V77, A:R78, A:S79, A:S80, A:S81, A:R82, A:T83, A:P84, A:S85, A:P96, A:R107, A:A109, A:N110 | 63 | 0.674 |
|  | 5 | A:R214, A:D216, A:Y217 | 3 | 0.632 |
| TNF-CPP-CD70  [Homo sapiens] | 1 | A:M1, A:S2, A:T3, A:E4, A:S5, A:M6, A:I7, A:R8, A:D9, A:V10, A:E11, A:L12, A:A13, A:E14, A:E15, A:A16, A:L17, A:P18, A:K19, A:T21, A:G22, A:G23, A:P24, A:Q25, A:G26, A:S27, A:R28, A:R29, A:C30, A:L31, A:F32, A:L33, A:S34, A:L35, A:F36, A:S37, A:F38, A:L39, A:I40, A:V41, A:A42, A:G43, A:A44, A:T45, A:T46, A:L47, A:F48, A:C49, A:L50, A:L51, A:H52, A:F53, A:G54, A:V55, A:I56, A:G57, A:P58, A:Q59, A:E61, A:E62, A:F63, A:P64, A:R65, A:D66, A:L67, A:S68, A:L69, A:I70, A:S71, A:P72, A:Q75, A:A76, A:S79, A:R82, A:R107, A:T319, A:L320, A:A321, A:A322, A:Y323, A:T324, A:T325, A:A326, A:S327, A:H330, A:P331, A:T332, A:A356, A:A357, A:Y358 | 90 | 0.755 |
|  | 2 | A:N95, A:P96, A:Q97, A:A98, A:E99, A:G100, A:Q101, A:L102, A:Q103, A:F140, A:K141, A:G142, A:Q143, A:G144, A:C145, A:P146, A:S147, A:T148, A:H149, A:V150, A:L151, A:L152, A:I173, A:K174, A:S175, A:P176, A:C177, A:Q178, A:R179, A:E180, A:T181, A:P182, A:E183, A:G184, A:A185, A:E186, A:A187, A:K188, A:P189, A:W190, A:Y191, A:E192, A:P193, A:R214, A:P215, A:D216, A:Y217, A:L218, A:D219, A:A221, A:E222 | 51 | 0.727 |
|  | 3 | A:Q256, A:A257, A:Q260, A:L261, A:P262, A:L263, A:E264, A:S265, A:G267, A:W268, A:Y271, A:Q293, A:G294, A:G295, A:P296, A:A297, A:L298, A:G299, A:R300, A:S301, A:L303, A:A304, A:A305, A:Y306, A:H307, A:R308, A:D309, A:G310, A:I311, A:Y312, A:V314, A:A339, A:A340, A:Y341, A:S342, A:P343, A:A344, A:S345, A:R346, A:S347, A:I348, A:S349, A:L350, A:S362, A:L363, A:L364, A:R365, A:L366, A:S367, A:F368, A:A369, A:A370, A:Y371, A:G372, A:W373, A:D374, A:V375, A:G407, A:R408, A:S409 | 60 | 0.694 |
